# Generation of developmentally competent oocytes and fertile mice from parthenogenetic embryonic stem cells

**DOI:** 10.1007/s13238-021-00865-4

**Published:** 2021-11-30

**Authors:** Chenglei Tian, Linlin Liu, Ming Zeng, Xiaoyan Sheng, Dai Heng, Lingling Wang, Xiaoying Ye, David L. Keefe, Lin Liu

**Affiliations:** 1 State Key Laboratory of Medicinal Chemical Biology, Nankai University, Tianjin, 300071 China; 2 Department of Cell Biology and Genetics, College of Life Sciences, Nankai University, Tianjin, 300071 China; 3 Department of Obstetrics and Gynecology, NYU Langone Health, 550 First Avenue, New York, NY 10012 USA; 4 Institute of Translational Medicine, Tianjin Union Medical Center, Nankai University, Tianjin, 300000 China

**Keywords:** parthenogenetic embryonic stem cells, primordial germ cell-like cells, imprinting, meiosis, oocytes

## Abstract

Parthenogenetic embryos, created by activation and diploidization of oocytes, arrest at mid-gestation for defective paternal imprints, which impair placental development. Also, viable offspring has not been obtained without genetic manipulation from parthenogenetic embryonic stem cells (pESCs) derived from parthenogenetic embryos, presumably attributable to their aberrant imprinting. We show that an unlimited number of oocytes can be derived from pESCs and produce healthy offspring. Moreover, normal expression of imprinted genes is found in the germ cells and the mice. pESCs exhibited imprinting consistent with exclusively maternal lineage, and higher X-chromosome activation compared to female ESCs derived from the same mouse genetic background. pESCs differentiated into primordial germ cell-like cells (PGCLCs) and formed oocytes following *in vivo* transplantation into kidney capsule that produced fertile pups and reconstituted ovarian endocrine function. The transcriptome and methylation of imprinted and X-linked genes in pESC-PGCLCs closely resembled those of *in vivo* produced PGCs, consistent with efficient reprogramming of methylation and genomic imprinting. These results demonstrate that amplification of germ cells through parthenogenesis faithfully maintains maternal imprinting, offering a promising route for deriving functional oocytes and having potential in rebuilding ovarian endocrine function.

**Supplementary Information:**

The online version contains supplementary material available at 10.1007/s13238-021-00865-4.

## INTRODUCTION

In most mammalian species, follicular reserve is determined at birth, and progressively declines with age. Both age and gonadotoxic chemo- or radio-therapies accelerate oocyte and follicular attrition and promote premature ovarian aging and infertility (Nagaoka et al. 2012; Grive and Freiman 2015; Zhang and Liu 2015). Augmenting the supply of oocytes could preserve the endocrine functions. Encouragingly, oocytes can be generated from embryonic stem cells (ESCs) derived from fertilized embryos or induced pluripotent stem cells (iPSCs) from somatic cells (Hayashi et al. 2012; Hikabe et al. 2016).

Parthenogenetic embryos, formed from activation and diploidization of oocytes, do not contain a paternal genome, such that parthenotes arrest at mid-gestation from defects in genomic imprinting and placental malfunction (Barton et al. 1984; Surani et al. 1984; Barlow and Bartolomei 2014). Despite their developmental incompetence, parthenogenetic embryonic stem cells (pESCs) can be generated from parthenogenetic embryos, and therefore allay ethical concerns associated with destruction of viable embryos (Sousa and Wilmut 2007). pESCs are produced from oocytes by chemical activation e.g., with strontium (Sr^2+^) (Swann and Ozil 1994; Moses and Kline 1995; Liu et al. 2002; Chen et al. 2009), and undergo dramatic epigenetic reprogramming and global demethylation (Li et al. 2009). The pESCs are minimally immunogenic to those who donate the founding oocytes (Kim et al. 2007; Didie et al. 2013; Espejel et al. 2014). Moreover, pESCs develop significantly fewer *de novo* coding mutations than do nuclear transfer ESCs and iPSCs (Johannesson et al. 2014). The utility of iPSCs is especially constrained by accumulation of somatic mutations and variability of differentiation (Blasco et al. 2011; Gore et al. 2011; Gao et al. 2015; Tapia and Scholer 2016; Kilpinen et al. 2017; Yoshihara et al. 2017; D'Antonio et al. 2018). Additionally, given that telomere reserve is critical for genomic stability and formation of germline (Bonis et al. 2014), telomeres of pESCs are comparable to those of ESCs (Yin et al. 2014). However, it remains unclear whether functional oocytes can be generated from pESCs and whether the aberrant imprinting of parthenogenetic embryos can be reprogrammed during formation of pESCs and differentiation into PGCLCs and oocytes.

## RESULTS

### Parthenogenetic ESCs (pESCs) acquire germline competence and maintain maternally expressed genes

**Figure 1:**
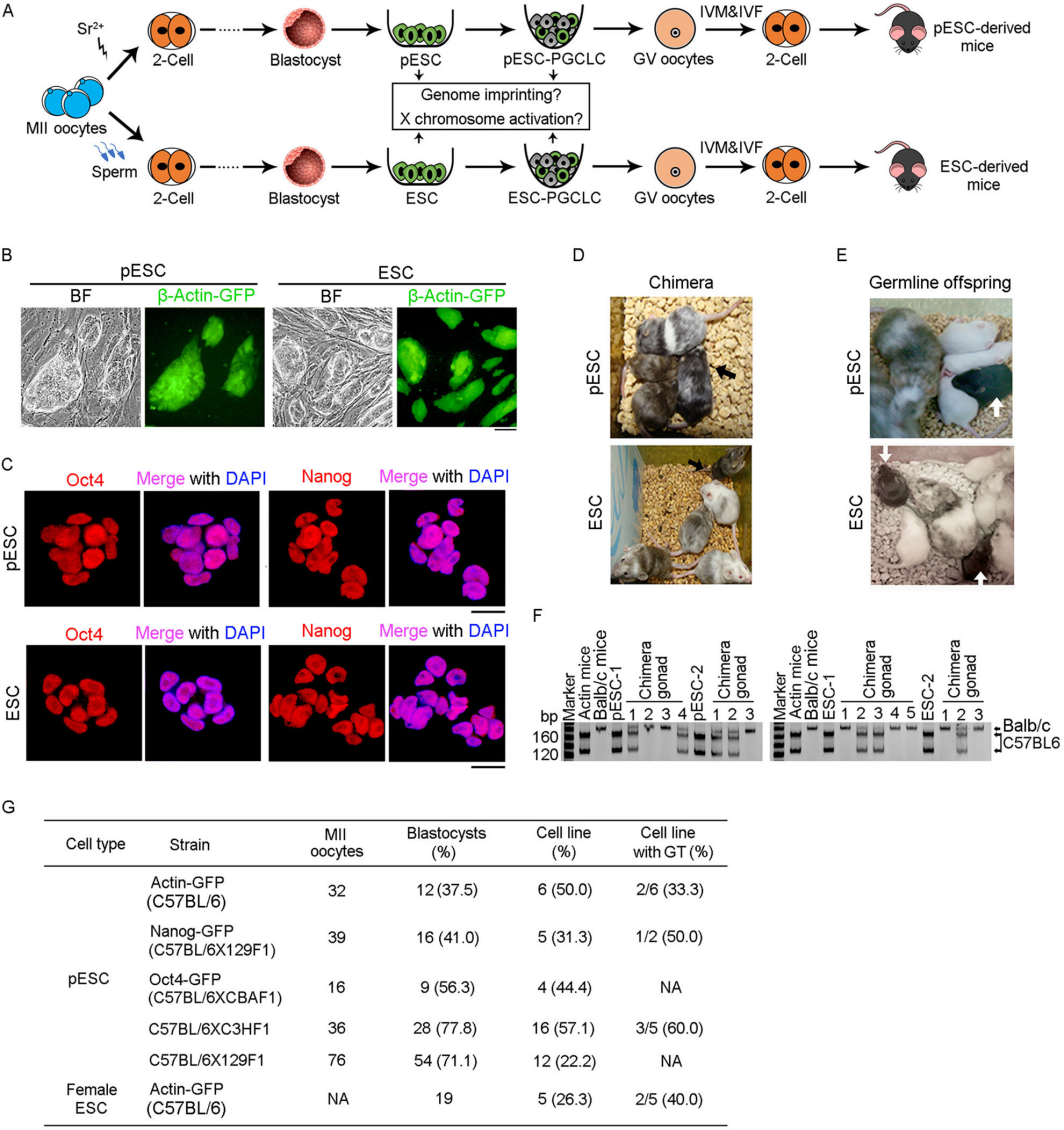
**Parthenogenetic ESCs (pESCs) acquire germline competence**. (A) Schematic summary illustrating the strategy of augmenting oocytes by derivation of pESCs from parthenogenetically activated oocytes or ESCs from fertilized embryos that are directed to differentiate into germ cells and functional oocytes. IVM, *in vitro* maturation; IVF, *in vitro* fertilization. (B) Morphology and β-Actin-GFP expression of pESCs and ESCs. Scale bar = 100 μm. (C) Expression of pluripotency markers (Oct4 and Nanog) of pESCs and ESCs. Scale bar = 20 μm. (D and E) Chimera (D) and germline competency (E) of pESCs and ESCs by 4–8-cell embryo injection. Germline offspring was produced by mating of chimeras with albino ICR mice. Albino Balb/c mice served as embryo donors, and pseudo-pregnant albino Kunming mice as surrogate mother. (F) Genotyping analysis of gonad in pESC (left) and ESC (right) derived chimeras generated from pESCs or ESCs by microsatellite primers D12Mit136. (G) Summary of derivation of pESCs and female ESCs from various mouse strains. Actin-GFP and Oct4-GFP pESCs were from *in vivo* (IVO) MII oocytes; Nanog-GFP pESCs from *in vitro* maturation (IVM) MII oocytes; C57BL/6XC3HF1 and C57BL/6X129F1 pESCs from IVO and IVM oocytes. NA, not available; GT, germline transmission competence; the efficiency (%) of GT cell lines is shown as the number of cell lines with germline transmission competence/number of cell lines tested

We generated parthenogenetic ESCs (pESCs) from β-Actin-GFP parthenogenetic embryos, and female (XX) ESCs from the same genetic background, to serve as controls (Fig. 1A). After seven days in culture, the outgrowths from parthenogenetic blastocysts exhibited morphology and size comparable to those of blastocysts developed from fertilized embryos, and expressed Actin-GFP (Fig. S1A). Morphology and expression of the pluripotent marker genes, Oct4 and Nanog in pESCs resembled those of ESCs (Fig. 1B and 1C). Both pESCs and ESCs generated chimeras with germline competence, as determined by the 4–8-cell embryo injection assay (Fig. 1D–F). Various pESC lines obtained from MII oocytes demonstrated germline competence (Fig. 1G). pESC lines generated from different mouse strains exhibited variability in the efficiency of cell line derivation from blastocysts, from 22% to 57%. The efficiency of germline transmission ranged from 33% to 60% (Fig. 1G). Of five female ESC lines derived from 19 blastocysts, two displayed germline competence (Fig. 1G). At passage 10, over 65% of chromosome spreads from pESCs displayed the normal complement of 40 chromosomes (Fig. S1B), similar to ESCs. These results demonstrate that pESCs derived from parthenogenetically activated oocytes exhibit pluripotency and developmental potential comparable to female ESCs.

**Figure 2:**
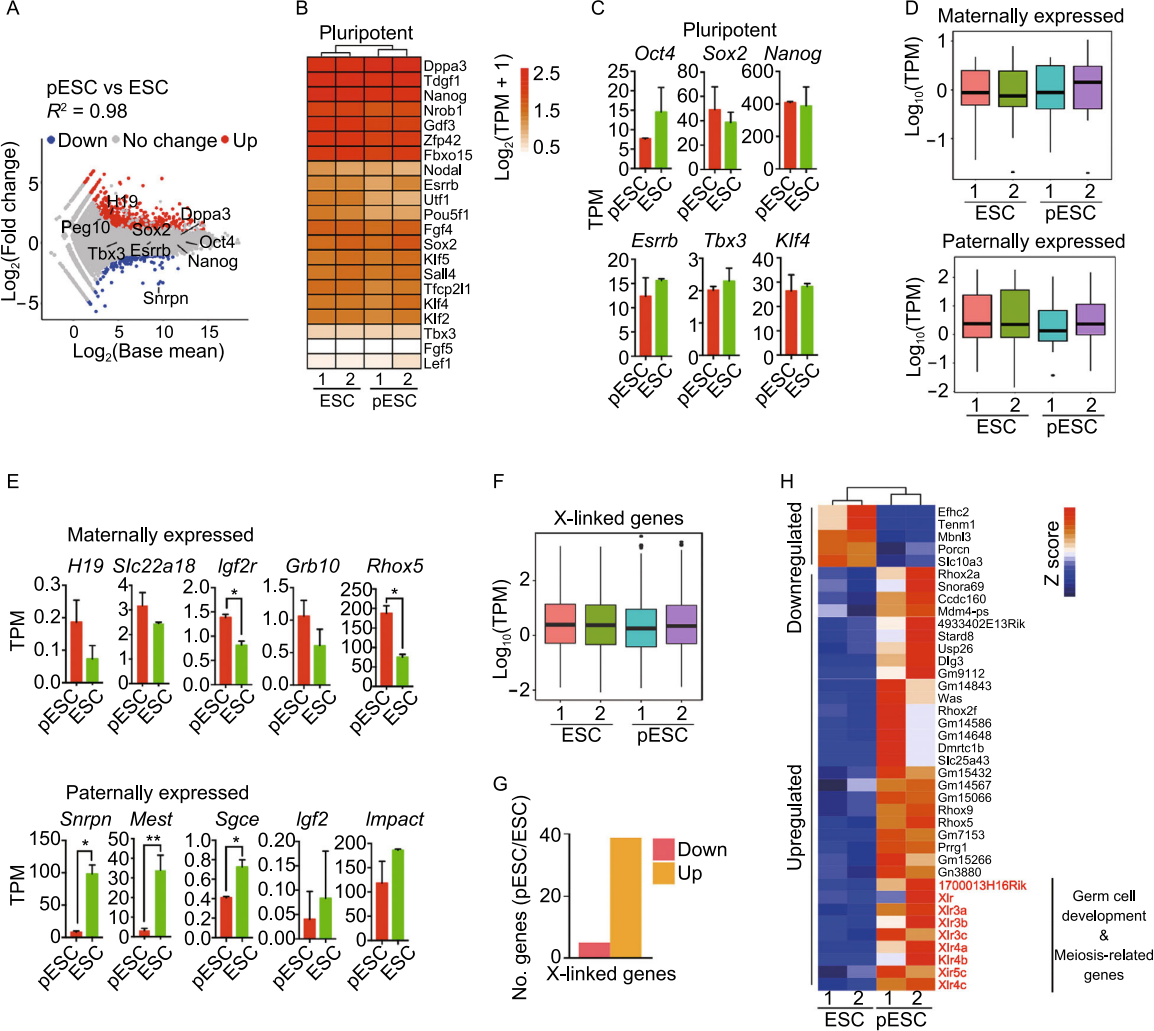
**Parthenogenetic ESCs (pESCs) maintain expression of maternal genes**. (A) Scatter-plots showing the differential gene expression profile between pESCs and ESCs. Two-fold changes (*P* < 0.05) are set as threshold. (B) Heatmap displaying similar expression profile of pluripotency genes between pESCs and ESCs. (C) Comparison of expression of representative pluripotency genes in pESCs and ESCs by RNA-seq. Data shown as mean ± SEM. (D) Box plot showing higher levels of maternally expressed genes but lower levels of paternally expressed genes in pESCs than in ESCs. (E) Expression of representative maternally and paternally expressed genes in pESCs and ESCs by RNA-seq. Data shown as mean ± SEM. (F) Box plot displaying expression profile of all X-linked genes in pESCs compared with that of ESCs. (G) More upregulated than downregulated X-linked genes in pESCs compared with ESCs. The threshold of differentially expressed genes (DEGs) is ≥ 2-fold (*P* < 0.05). (H) Typical upregulated and downregulated X-linked genes of pESCs compared with ESCs (≥ 2 fold change, *P* < 0.05). Genes in red, germ cell development or meiosis. All RNA-seq analysis represents two biological replicates

To characterize the molecular basis underlying their pluripotency, we employed RNA-seq analysis to compare the transcriptional profiles of pESCs to female ESCs. Genome-wide gene expression profile was strikingly similar (*R*^2^ = 0.98), but not identical between pESCs and ESCs (Fig. 2A). Like ESCs, pESCs expressed key pluripotency genes, including *Oct4*, *Sox2*, and *Nanog,* at high levels. Expression levels of naive marker genes also were similar between pESCs and ESCs (Fig. 2B and 2C). Expression levels of maternal genes, such as *H19*, *Slc22a18*, *Grb10*, *Igf2r* and *Rhox5*, in pESCs exceeded those of ESCs (Fig. 2D and 2E). Expression levels of paternal genes including *Igf2*, *Snrpn*, *Mest*, *Impact*, and *Sgce* were lower in pESCs than in ESCs (Fig. 2D and 2E), consistent with the maternal origin of pESCs. Comparison by GO analysis of upregulated genes between pESCs and ESCs showed enrichment for meiotic cell cycle and germ cell development in pESCs (Figs. S1C and S1D). X-linked genes were expressed at similar levels in pESCs and ESCs (Fig. 2F), but more upregulated than downregulated genes were found in X-chromosomes from pESCs (Fig. 2G). Upregulated genes from pESCs were enriched for those involved in germ cell development and meiosis (e.g., *Xlr*, *Xlr3a*, *Xlr3b*, *Xlr3c*, *Xlr4a*, *Xlr4b*, *Xlr4c*, *Xlr5c*, *1700013H16Rik* etc.) (Fig. 2H). These results imply that both ESCs and pESCs have two active X chromosomes, but comparatively more X-linked genes are upregulated indicative of higher X chromosome activation in pESCs than in ESCs. X chromosome re-activation represents important molecular criteria for naïve pluripotency of female ESCs (Huang et al. 2014; Pasque et al. 2014; Theunissen et al. 2016). Also, germ cells derived by differentiation of embryoid bodies (EBs) from pESCs exhibit higher expression of germ cell genes than those of ESCs (Liu et al. 2011).

### Comparison of pESC-derived PGCLCs with embryonic PGCs at global transcriptional levels

**Figure 3:**
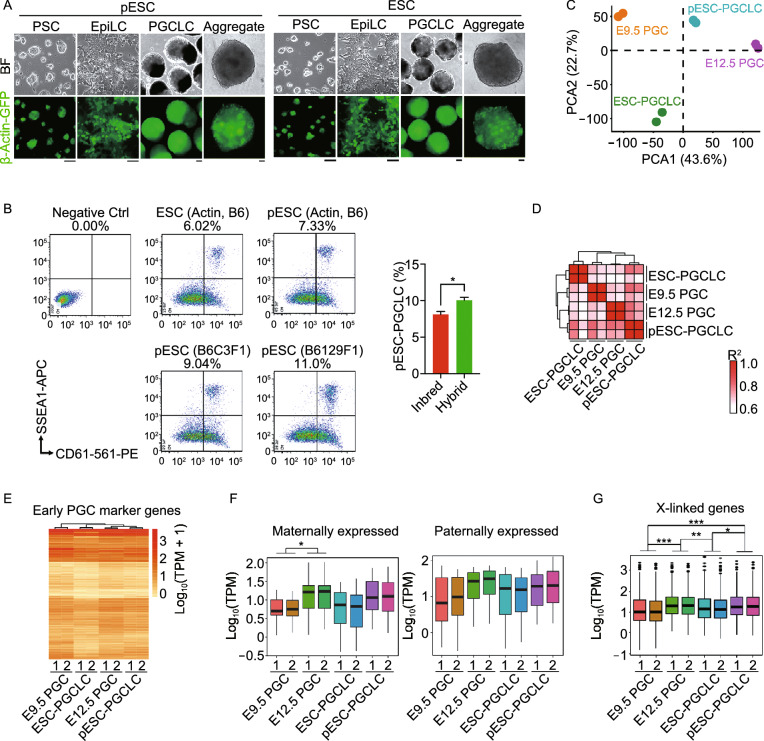
**Comparison of pESC-PGCLCs and PGCs in vivo at global transcription levels and in imprinting**. (A) Epiblast-like cells (EpiLCs) and PGCLCs induced from pESCs or ESCs bearing Actin-GFP, and aggregates of PGCLCs with E12.5 gonad somatic cells sorted from albino ICR mice for one day. Scale bar = 50 μm. (B) Representative PGCLCs induction (left) from pESCs or ESCs from Actin-GFP C57BL/6 mice, or pESCs from hybrid mice (C57BL/6XC3HF1 or C57BL/6X129F1) by FACS, and statistics (right) of PGCLCs induction efficiency from inbred (Actin, B6) and hybrid pESCs. Three replicates for inbred ESCs and pESCs and two replicates for hybrid pESCs. SSEA1 and CD61 double-positive cells represent PGCLCs. Absence of the antibody served as negative control. (C) PCA analysis by RNA-seq of E9.5 PGCs, E12.5 PGCs and pESC- or ESC-PGCLCs. (D) Pearson’s correlation coefficient (*R*^2^) graph of E9.5 PGCs, E12.5 PGCs, pESC-PGCLCs and ESC-PGCLCs. The value of 1.0 represents perfect positive correlation and 0 represents no correlation between the two samples. (E) Heatmap showing the expression profile of early PGC marker genes in E9.5 PGCs, E12.5 PGCs, ESC-PGCLCs and pESC-PGCLCs. Gene expression level is shown as log_10_(TPM + 1). (F) Box plot displaying expression profile of maternal and paternal genes in E9.5 PGCs, E12.5 PGCs, and pESC- or ESC-PGCLCs. (G) Box plot displaying expression profile of X chromosome-linked genes in E9.5 PGCs, E12.5 PGCs, and pESC- or ESC-PGCLCs. Data shown as Mean ± SEM. *P < 0.05, **P < 0.01, ***P < 0.001. All RNA-seq analysis represents two biological replicates

To test the hypothesis that pESCs could provide a rich source of germ cell induction and functional oocytes, we induced PGCLCs from pESCs and compared them to PGCLCs from XX ESCs, following the protocol described previously for induction of PGCLCs from ESCs and iPSCs (Hayashi et al. 2012; Hayashi and Saitou 2013). PGCLCs derived from XX ESCs and pESCs also were compared to embryonic PGCs *in vivo*. pESCs and XX ESCs from the same inbred genetic background formed epiblast-like cells (EpiLCs) and PGCLCs (Fig. 3A) at similar efficiencies (8.12% ± 0.56% for pESC-PGCLCs and 6.50% ± 1.24% for ESC-PGCLCs) by FACS (SSEA1 and CD61 double positive cells indicative of PGCLCs) (Hayashi et al. 2012; Hikabe et al. 2016) (Fig. 3B). Absence of the antibodies served as negative control. pESCs from hybrid genetic background exhibited higher efficiency in the induction of PGCLCs (9.58% ± 0.54% in C57BL/6XC3HF1 pESCs and 10.53% ± 0.47% in C57BL/6X129F1 pESCs), compared to those of inbred pESCs (*P* < 0.05) (Fig. 3B). To understand the underlying molecular changes in the PGCLCs derived from pESCs, we performed RNA-seq analysis of these cells and compared them to those of ESCs and ESC-PGCLCs. Broadly, transcriptome profile between pESC-PGCLCs and ESC-PGCLCs was similar, yet some differences were found (Fig. S2A and S2B). pESCs/ESCs and their respective PGCLCs were readily distinguishable at genome-wide level (Fig. S2B–D), consistent with previous findings (Zhou et al. 2016). Compared with those of pESCs or ESCs by KEGG analysis, most upregulated genes in pESC-PGCLCs and ESC-PGCLCs were enriched in signaling pathways regulating pluripotent stem cells (PSC), TGF-β and Wnt (Fig. S2D and S2E) (Ohinata et al. 2009; Zhou et al. 2016). By GO analysis, the processes of reproductive system development and meiotic cell cycle were activated in both pESC- and ESC-PGCLCs (Fig. S2D and S2F). Global gene expression was comparable among pESC-PGCLCs, ESC-PGCLCs, and E12.5 PGCs. The correlation coefficient (*R*^2^) between pESC-PGCLCs and ESC-PGCLCs was 0.70 (Fig. S2G).

We further compared the transcriptome of pESC- or ESC-PGCLCs to that of E9.5 and E12.5 PGCs *in vivo* by PCA and clustering analysis. The global transcriptional profile of each sample can be clearly defined and separated (Fig. 3C and 3D). Based on the current induction methods, PGCLCs generated from ESCs or iPSCs resemble PGCs in their increased expression of early germ cell marker genes (Hayashi et al. 2012; Ohta et al. 2017; Hackett et al. 2018). Notably, the early PGC marker gene expression pattern, based on the published gene list (Miyauchi et al. 2017), was similar among pESC-PGCLCs, ESC-PGCLCs, and E9.5 and E12.5 PGCs (Fig. 3E).

### DNA methylation in pESCs and ESCs as well as in PGCLCs derived from pESCs or ESCs and PGCs *in vivo*

An important aspect of germ cell development is erasure of genomic imprints in PGCs. Erasure of imprints begins in migrating PGCs and is essential for forming functional germ cells (Seisenberger et al. 2012; Kagiwada et al. 2013; Miyoshi et al. 2016). Differences in DNA methylation between maternal and paternal alleles of imprinted genes are laid down in a sex specific manner in male and female gametes (Davis et al. 2000; Lucifero et al. 2002). For instance, PGCs undergo demethylation in *Igf2r* and *H19* genes (Geijsen et al. 2004). We analyzed expression profiles of imprinted genes in pESC- and ESC-PGCLCs and compared them to E9.5 and E12.5 PGCs. Overall expression levels of maternal and paternal genes did not differ significantly between pESC-PGCLCs and ESC-PGCLCs (Fig. 3F). These results demonstrate that imprinted genes are increasingly expressed during differentiation of PSCs into PGCLCs, corroborating significant demethylation during PGC development, as reported previously (Miyoshi et al. 2016; Meyenn et al. 2016).

X chromosomes gradually reactivate in PGCs from E9.5 to E12.5 (S. M. Chuva de Sousa Lopes et al. 2008; Seisenberger et al. 2012). In line with this, expression levels of X-linked genes in E9.5 PGCs appeared to be lower than those of E12.5 PGCs as well as of pESC-PGCLCs or ESC-PGCLCs (Fig. 3G). These data indicate that PGCLCs differentiated from pESCs or ESCs exhibit similar high rates of X-chromosome activation, resembling E12.5 PGCs.

Also, we compared DNA methylation profile of pESCs and ESCs by reduced representation bisulfite sequencing (RRBS). Genome-wide DNA methylation profile revealed very similar DNA methylation landscapes between pESCs and ESCs (Fig. S3A and S3B). Furthermore, we explored the DNA methylation profiles of X chromosomes and imprinted genes. The overall DNA methylation levels on X chromosomes in pESCs resembled that of XX ESCs (Fig. S3C). Parental imprinting is a heritable epigenetic mechanism resulting in parent-specific monoallelic expression of a subset of genes. DNA methylation levels of maternally expressed (paternally imprinted) genes did not differ between pESCs and ESCs, but DNA methylation levels of paternally expressed (maternally imprinted) genes (e.g., *Snrpn*, *Impact*, *Mest*, and *Sgce*) were higher in pESCs than in ESCs (Fig. S3D and S3E). Higher methylation of some maternally imprinted genes in pESCs mirrors their decreased expression levels (Figs. 2E and S3E).

**Figure 4:**
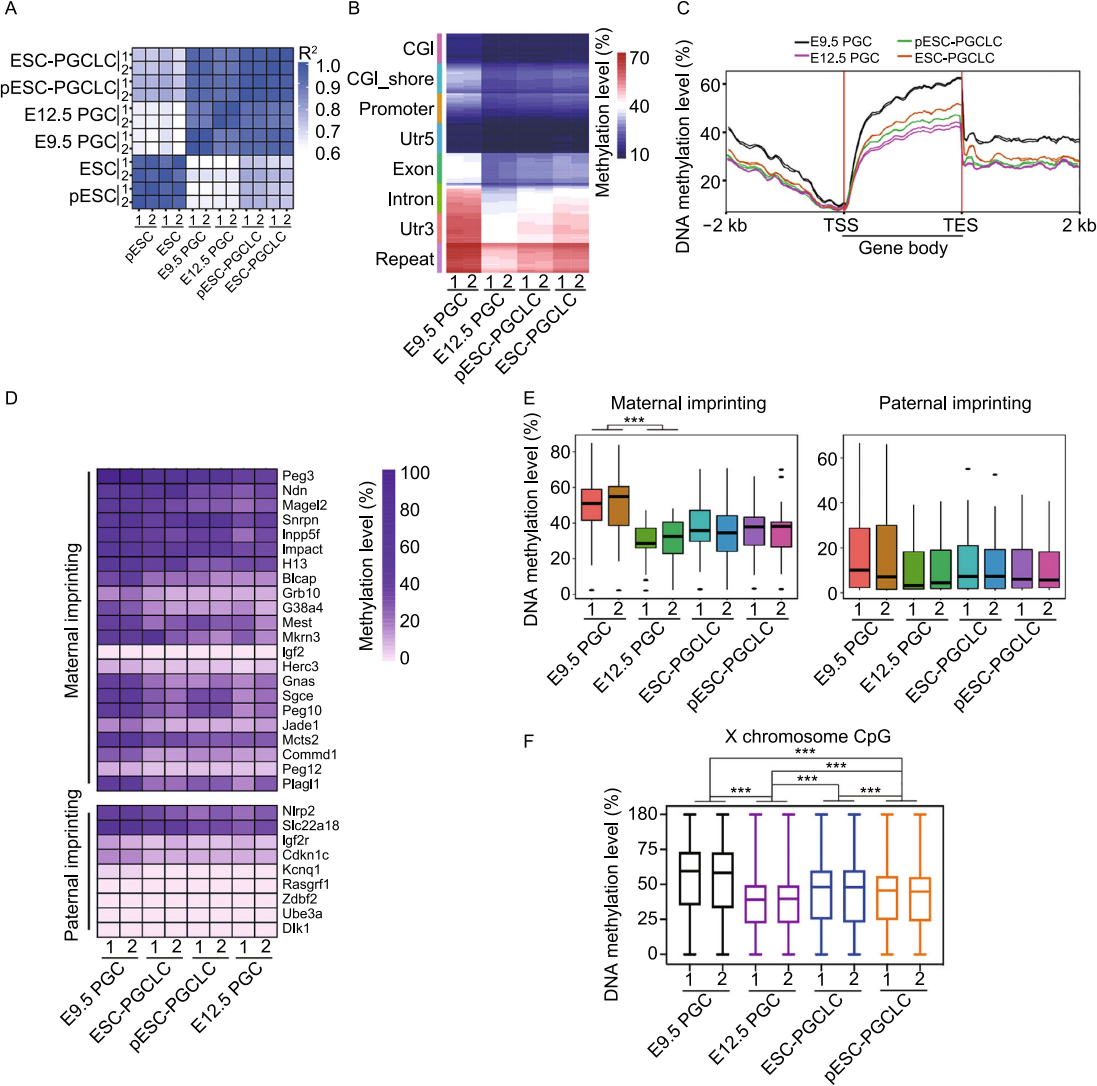
**Comparison of pESC-PGCLCs and PGCs at DNA methylation levels**. (A) Correlation factors of genome-wide DNA methylation levels in E9.5 PGCs, E12.5 PGCs, ESC-PGCLCs and pESC-PGCLCs as well as ESCs and pESCs. (B) Heatmap showing genome-wide DNA methylation levels at different gene regions of E9.5 PGCs, E12.5 PGCs, ESC-PGCLCs and pESC-PGCLCs. (C) Genome-wide DNA methylation level in gene body regions including up- and down-stream 2 kb of gene body. (D) Methylation level of maternal and paternal imprinting genes in pESC-PGCLCs, ESC-PGCLCs, E9.5 PGCs and E12.5 PGCs. Methylation counts are provided in Table S1. (E) Box plot showing DNA methylation levels of maternal and paternal imprinting in pESC-PGCLCs, ESC-PGCLCs, E9.5 PGCs and E12.5 PGCs. (F) Box plot showing global DNA methylation levels of X chromosomes in pESC-PGCLCs, ESC-PGCLCs, E9.5 PGCs and E12.5 PGCs. Data shown as mean ± SEM. *P < 0.05, **P < 0.01; ***P < 0.001. RRBS analysis represents two biological replicates

After we established the similarly high quality of those pES cell lines and were confident about their germline capacity by chimera production assay, we focused on directed differentiation *in vitro* of PGCLCs from pESCs or ESCs, and compared the methylome of PGCLCs from pESCs or ESCs with that of *in vivo* E9.5 or E12.5 PGCs. ESC-PGCLCs, pESC-PGCLCs, E9.5 PGCs and E12.5 PGCs *in vivo* exhibited strikingly similar global methylation patterns (*R*^2^ > 0.90) (Fig. 4A). Global methylation levels of naive ESCs/pESCs generally were similar to those of PGCs *in vivo* (Fig. S4A and S4B)*,* like previous reports, although EpiLCs displayed notably higher methylation levels (Meyenn et al. 2016; Shirane et al. 2016). We did not measure the methylation levels of EpiLCs during PGCLC induction. The methylation levels in different regions of the genome, including GCI, promoter, Utr5, Exon, etc., were lower in E12.5 PGCs, pESC-PGCLCs and ESC-PGCLCs than in E9.5 PGCs (Figs. 4B, S4A and S4B). Also, global methylation levels of gene body regions (± 2 kb, including TSS and TES) were lower in E12.5 PGCs, pESC-PGCLCs and ESC-PGCLCs than in E9.5 PGCs (Fig. 4C). These data demonstrate that despite heterogeneity of cell lines, PGCLCs induced from PSCs generally mimic PGCs *in vivo* (Hajkova et al. 2002; Seisenberger et al. 2012), also consistent with previous findings of methylome dynamics of PGCLC induction from PSCs (Meyenn et al. 2016; Shirane et al. 2016).

Methylation also regulates expression of imprinted genes in germ cells (Reik et al. 2001). The transcriptional profile of PGCs is tightly controlled, despite global hypomethylation, and sequences that carry long-term epigenetic memory (imprints, CpG islands on the X chromosome, and germline-specific genes) become demethylated in late PGCs (Seisenberger et al. 2012; SanMiguel and Bartolomei 2018). DNA methylation levels of most imprinted genes in E9.5 PGCs exceeded those of E12.5 PGCs as well as of pESC- and ESC-PGCLCs (Fig. 4D and 4E; Table S1), implying that loss of imprinting takes place during PGC development (Meyenn et al. 2016). DNA methylation levels of maternally and paternally imprinted genes in pESC-PGCLCs broadly were similar to those of ESC-PGCLCs and also PGCs *in vivo* (Fig. 4D and 4E; Table S1). Moreover, overall DNA methylation levels on X chromosomes in E12.5 PGCs were lower than those of E9.5 PGCs (Fig. 4F). Indeed, the majority of PGCs entering the genital ridge at E10.5–13.5 days reactivate their X chromosomes (Tam et al. 1994). The overall methylation levels of X chromosomes in pESC-PGCLCs turned to be more similar to those of ESC-PGCLCs and E12.5 PGCs than of E9.5 PGCs (Fig. 4F). These results suggest that the genome of pESC-PGCLCs is relatively hypomethylated, with two active X chromosomes.

### Oocytes regenerated from pESCs produce fertile offspring

**Figure 5:**
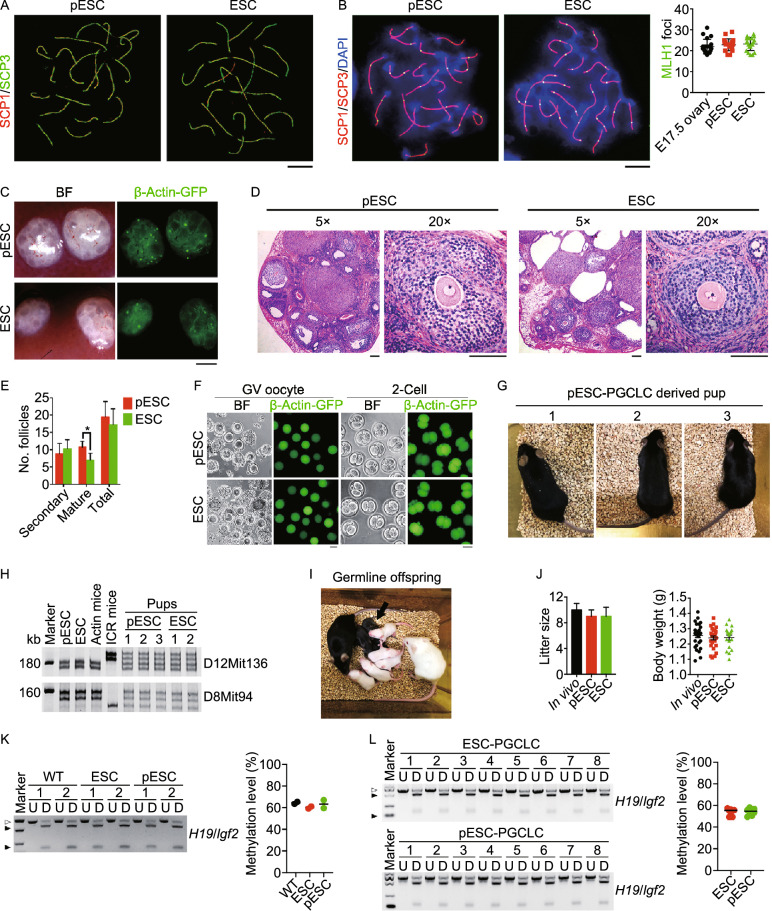
**Fertile mice produced from oocytes of pESCs**. (A) Normal synaptonemal complex in pESC-and ESC-derived meiocytes 5 days following transplantation of the PGCLCs aggregates based on pachytene spread by super high-resolution microscopy (SIM). Scale bar = 5 μm. (B) Statistics of MLH1 foci per cell at pachytene stage in meiocytes derived from pESC- or ESC-PGCLCs. Scale bar = 5 μm. (C) Morphology and Actin-GFP expression of reconstituted ovaries. Scale bar = 1 mm. (D) Folliculogenesis of pESC- and ESC-derived reconstituted ovaries by H&E staining. Scale bar = 100 μm. (E) Follicle count of pESC- and ESC-reconstituted ovaries 28 days following transplantation of the aggregates into kidney capsule. Data shown as Mean ± SEM (*n* = 4). *P < 0.05. (F) GV oocytes isolated from pESC- and ESC-reconstituted ovaries, and 2-cell embryos after IVM and IVF. Scale bar = 50 μm. (G) Healthy adult mouse produced from pESC-PGCLCs derived oocytes following IVM and IVF. (H) Genotyping analysis of the pups by microsatellite primers D8mit94 and D12Mit136. DNA was isolated from tail tip tissues. (I) Offspring of pESC-PGCLCs derived mice by mating with albino ICR mice. (J) Similar litter size and body weight of pups produced from oocytes of pESCs and ESCs in comparison with those of wild-type (/*in vivo*) mice served as a control. (K) Combined bisulfite restriction analysis (COBRA) of typical imprints *H19*/*Igf2* of tail tissue from ESC- and pESC-PGCLC derived mice (*n* = 2). Wild-type (WT) mice from normal breeding at the same background served as control. PCR products were either digested (D) or undigested (U) with the respective enzyme. The digested and undigested fragments are indicated by black and white triangles, respectively. Right panel, Methylation level analysis of COBRA by Image J. (L) COBRA of typical imprints *H19*/*Igf2* of the tail tissue from the offspring derived from ESC- and pESC-PGCLC derived mice (*n* = 8). Right panel, Methylation level analysis of COBRA by Image J (*n* = 8)

Moreover, we tested whether PGCLCs, differentiated from pESCs, can produce developmentally competent oocytes. Meiosis and oocyte growth were induced by aggregation of PGCLCs with fetal E12.5 gonadal somatic cells, including pre-granulosa cells, followed by transplantation of the aggregates into the kidney capsule, to form reconstituted ovaries (Shen et al. 2006; Qing et al. 2008). Rock inhibitor and Vitamin C were supplemented in the aggregation medium to improve follicular development of PGCLCs aggregated with fetal gonadal somatic cells (Tian et al. 2019). To evaluate meiotic progression of PGCLCs derived from pESCs, we analyzed homologous chromosome pairing and synapsis, by immunofluorescence microscopy of SCP1/3 elements, and recombination by MLH1, key events of meiotic prophase I (Handel et al. 2014). SCP1 and SCP3 together form an evolutionarily conserved protein structure, the synaptonemal complex (SC), located along the paired meiotic chromosomes, which plays an important role in meiosis (Yuan et al. 2000). MLH1 is a mismatch repair gene involved in meiotic crossing over and recombination (Baker et al. 1996; Edelmann et al. 1996). Aggregates were treated with 3 μmol/L retinoic acid (RA) for one day, then transplanted into kidney capsules of oophorectomized females for five days, to facilitate PGCLCs entry into meiosis, similar to meiocytes of E16.5–E17.5 female gonads. PGCLCs aggregated with E12.5 gonad somatic cells differentiated into meiocytes with discernible SCP3 elements, which indicate homologous pairing. In contrast, pseudo-aggregates formed from only E12.5 gonad somatic cells as negative controls, displayed only background staining of some green spots but no distinct SCP3 lateral elements (Fig. S5A). SCP1/3 stained the axial elements of synaptonemal complexes at leptotene, zygotene, pachytene, and diplotene stages of prophase I, similar to that observed in ESC-PGCLCs (Figs. 5A and S5B). MLH1 foci appeared at crossover sites, with a frequency similar to E17.5 meiocytes and PGCLCs differentiated from ESCs (Fig. 5B). Hence, PGCLCs derived from pESCs can undergo normal homologous pairing and recombination during meiotic progression.

To further validate the germ cell identity of PGCLCs originating from pESCs, transplantation of aggregated PGCLCs into kidney capsules for 4 days revealed Actin-GFP fluorescence co-localized with cytoplasmic Dazl staining, typical germ cell marker (Fig. S6A). Twenty-eight days after transplantation of aggregates, Actin-GFP fluorescence within follicles was visible in reconstituted ovaries (Fig. 5C). Developing and mature follicles with germinal vesicle (GV) oocytes were observed in sections stained by H&E (Fig. 5D). Immunofluorescence staining of oocytes for Vasa, Dazl and GFP, and granulosa cells for Foxl2 and Gata4 further confirmed the cellular identify of reconstituted ovaries (Fig. S6B). The number of total follicles did not differ between reconstituted ovaries formed from pESC- or ESC-PGCLCs, but reconstituted ovaries from pESC-PGCLCs had more mature follicles than did ESC-PGCLCs (Fig. 5E).

We analyzed the function of reconstituted ovaries by measuring endocrine activity, including serum levels of follicle-stimulating hormone (FSH), estradiol (E2) and anti-Mullerian hormone (AMH) in the recipient NOD-SCID mice. The bilaterally oophorectomized (OE) mice, without receiving the ovarian grafts, served as negative controls. Serum FSH levels were reduced and E2 and AMH levels increased markedly in the OE recipients receiving the reconstituted ovarian grafts (Fig. S6C), indicating that PGCLCs derived from pESCs, following aggregation with fetal gonadal somatic cells, develop into functional follicles in reconstituted ovaries with capacity to restore the hormone production and secretion. Because the germ cells and accompanied somatic cells interact and both are essential for follicle development (Buccione et al. 1990), both germ cells and somatic cells contributed to the endocrine function. Hence, the follicles of the reconstituted ovaries from germ cell-like cells differentiated from pESCs recovered the endocrine functions of ovariectomized mice, providing further evidence of the functionality of the germ-cell like cells and the derived-oocytes.

Furthermore, to test whether oocytes generated from pESCs can produce fertile pups, we mechanically dissected GV oocytes from follicles in reconstituted ovaries and performed *in vitro* maturation (IVM) and *in vitro* fertilization (IVF). PGCLCs-derived oocytes were able to reach MII stage, fertilize, and cleave to two-cell embryos (Fig. 5F). Efficiency of cleavage to two-cells after IVM and IVF of pESC-PGCLCs-derived GV oocytes was 35.4% (64 two-cell embryos developed from IVM and fertilization of 181 GV oocytes), an efficiency comparable to that of ESC-PGCLCs (52 two-cell embryos from 147 GV oocytes) (Table S2). Three live pups were obtained following transfer of 64 two-cell embryos developed from oocytes of pESC-PGCLCs (Fig. 5G). The efficiency of embryo development to term from pESC-PGCLCs approximated half of PGCs *in vivo* which we recently reported using the same aggregation and transplantation methods (Sheng et al. 2019) (also, Table S2). The origin of offspring from PGCLCs was further validated by strain-specific microsatellite marker assays of donor and recipient mice (Fig. 5H). All mice survived and grew to healthy adulthood. Fertility and germline competence were normal in these animals (Fig. 5I). Litter size and body weight of offspring generated from oocytes of pESC-PGCLC-derived mice did not differ from those produced by *in vivo*, natural breeding, or from ESC-PGCLC-derived mice (Fig. 5J). Tissue samples such as heart, liver, spleen and gonad obtained from ESC- and pESC-PGCLC derived mice as well as the gonad from the offspring derived from ESC- and pESC-PGCLC derived mice expressed GFP fluorescence (Fig. S7A–C), further validating the origin of mice produced from donor ESCs/pESCs and their derived oocytes.

We also compared the imprinting status of mice produced from PGCLCs to that of normally bred mice. Methylation levels of the familiar imprinted genes, *H19*/*Igf2*, *Snrpn*, *Igf2r*, *Dlk1*/*Gtl2* and *Mest*, in PGCLC-derived mice and the gene expression levels did not differ from those of naturally bred, wild-type mice (Figs. 5K and S8A–C). Moreover, methylation levels of imprinted genes were comparable among second-generation pups (pups generated form PGCLC-derived mice after breeding) of pESC-PGCLCs and ESC-PGCLCs and normally bred mice (Figs. 5L, S8D and S8E). Together, PGCLCs differentiated from pESCs develop into fully functional oocytes and produce healthy, fertile offspring.

## DISCUSSION

We show that differentiation of the pESCs derived from parthenogenetically activated oocytes provides an unlimited source of developmentally competent oocytes, capable of producing fertile mice. Transcriptome, methylome, and imprinting patterns of pESC-derived PGCLCs closely resemble those of ESC-derived PGCLCs as well as *in vivo* produced PGCs.

Efficiency of generating pESCs from activated oocytes with strontium varies among mouse strains, ranging from about 20% in inbred strains (e.g., C57BL/6), comparable to derivation of inbred ESCs (Nagy et al. 1993; Eggan et al. 2001), to about 30% in hybrid strains. On the inbred C57BL/6 background, germline-competent pESC lines were obtained with stable XX chromosomes (Fig. S1B) from an average of one out of 16 oocytes. After expansion in culture for 10 passages, such one cell line could produce 2.4 × 10^6^ GV oocytes (Table S3). Recently, PGCLCs were successfully expanded *in vitro* to elevate their numbers (Ohta et al. 2017). BMP and retinoic acid synergistically induce PGCLCs derived from ESCs into fetal primary oocytes (Miyauchi et al. 2017). Vitamin C and Rock inhibitors promote aggregation, induction of meiosis, and oocyte and follicle development (Tian et al. 2019). Currently, the development and growth of pESC or ESC-derived PGCLCs as well as of PGCs *in vivo* following their transplantation are still limited. GV oocytes from pESCs and ESCs show similar maturation rate, but the efficiency of embryo development to term from pESC-PGCLCs is about half of PGCs *in vivo*. Although overall expression levels of maternal and paternal genes do not differ significantly between pESC-PGCLCs and ESC-PGCLCs and they also exhibit similar high rates of X-chromosome activation, subtle differences do exist in specific transcriptome and methylation between PGCLCs differentiated from pESCs or ESCs and PGCs *in vivo*. Future experiments must resolve these issues and increase the number of viable embryos available from PGCLCs or PGCs. Nevertheless, these oocytes can contribute to folliculogenesis and reconstitute endocrine function.

PGCLCs also have been successfully induced from human ESCs and iPSCs (Irie et al. 2015; Sasaki et al. 2015), and these hESCs/hiPSCs-derived PGCLCs can differentiate into oogonia (Yamashiro et al. 2018). Generation of oocytes *in vitro* from human PSCs has yet to be achieved. Additionally, a number of patients undergo failed embryonic development in clinical recurrent IVF/ICSI, because of defective maturation of GV oocytes. But parthenogenetic embryonic stem cells require matured oocytes, limiting pESC derivation in these patients. Encouragingly, human pESCs also have been derived by simple methods using chemical activation of oocytes. Human pESCs show epigenetic changes in imprinted genes compared with hESCs, including X chromosome activation, imprinted DMRs, distinct imprinting signatures and biased differentiation (Kim et al. 2007; Lin et al. 2007; Mai et al. 2007; Revazova et al. 2007; Stelzer et al. 2013; Sagi et al. 2016, 2019). Our data that PGCLCs differentiated from pESCs develop into fully functional oocytes and produce healthy, fertile mice with normal imprinting provides proof of principle, suggesting that it is also possible to generate oocytes from human pESCs and used for reconstructing ovarian functions. However, the safety on corresponding metabolic and development phenotypes of live pups needs to be critically evaluated in future experiments. Also, extensive research is needed to ensure that PGCLCs generated from pESCs resemble PGCs *in vivo* at molecular levels and the competent embryo development like that of IVF from normal oocytes. Clearly, additional work will be needed to extend our findings to humans. If successful in humans, this approach could amplify the severely limited stores of a woman’s oocytes, to potentially recover endocrine functions with age, and to produce autologous pluripotent stem cells essential for regenerative medicine. This strategy also may provide a technical advance to the ESC field by producing an unlimited number of oocytes for nuclear transfer ESCs.

## MATERIALS AND METHODS

### Animal care and use

Use of mice for this research was approved by the Nankai University Animal Care and Use Committee. All mice used in this study were taken care of and operated according to the relevant regulations. Mice were housed and cared in individually ventilated cages (IVCs) on a standard 12 h light:12 h dark cycle in the sterile Animal Facility at College of Life Sciences. C57BL/6-Tg (CAG-EGFP) C14-Y01- FM131Osb mice that carry β-Actin-GFP and Oct4-GFP (OG2) mice (C57BL6 X CBA) were obtained from Model Animal Research Center of Nanjing University. It was anticipated that Actin-GFP fluorescence could facilitate identification of transplant cells in transplantation experiments. 4–6-Week-old NOD-SCID mice (Female, #406), 6-week-old albino Balb/c mice (Female, #401), 6–8-week-old albino ICR mice (Male and female, #201) and 6-week-old albino Kunming (KM) mice (Female, #202) were purchased from Beijing Vital River Laboratory Animal Technology Co., Ltd.

### Isolation and primary cultured MEFs

MEFs were derived from E13.5 ICR mice embryos isolated by caesarean section and washed in phosphate-buffered saline (PBS). Heads and visceral tissues were removed, and remaining tissue washed in PBS, then submerged in 0.25% trypsin–EDTA (0.25% TE, Invitrogen) and incubated at 37 °C for 10 min. Tissue was pipetted repeatedly to aid dissociation, washed then dissociated cells added to MEF medium and plated (passage 0, P0). MEF medium contains DMEM (Invitrogen) supplemented with 10% Fetal Bovine Serum (FBS, Hyclone), 1 mmol/L L-glutamine (Invitrogen), 1% nonessential amino acid stock (NEAA, Sigma), and 50 units/mL penicillin and 50 μg/mL streptomycin (Invitrogen).

### Establishment of mouse pESC and ESC lines

Actin-GFP female mice at 6–8 weeks of age were superovulated with 5 IU pregnant mare serum gonadotrophin (PMSG), followed by 5 IU human chorionic gonadotropin (hCG) 46–48 h later. Oocytes enclosed in cumulus masses were collected from oviduct ampullae 14 h after hCG injection. *In vitro* maturation of GV oocytes 46 h following PMSG injection was achieved by culture in IVM medium following the method described (Eppig et al. 2009). Cumulus cells of MII oocytes were removed by pipetting, after a brief incubation in 0.03% hyaluronidase prepared in potassium simplex optimized medium (KSOM_AA_) containing 14 mmol/L HEPES and 4 mmol/L sodium bicarbonate (HKSOM). Oocytes were reliably activated by treatment for 4 h with 10 mmol/L SrCl_2_ and cytochalasin D prepared in Ca^2+^-free KSOM (Chen et al. 2009; Liu et al. 2011). Activated oocytes with two pronuclei were defined as diploid parthenotes and cultured in 50 μL droplets of pre-equilibrated KSOM, covered with mineral oil at 37 °C in a humidified atmosphere of 6% CO_2_ in air for 4–5 days. To obtain fertilized embryos, Actin-GFP females were superovulated and mated individually with Actin-GFP males of proven fertility. Oviducts of successfully mated females 3.5 days after mating were flushed with HKSOM using a Pasteur pipette, and blastocysts were obtained to derive normal ESC lines.

pESC and ESC lines were established and characterized in collaboration with D. Keefe based on the method described (Chen et al. 2009; Liu et al. 2011). Blastocysts were plated onto mitomycin C-treated MEF cells served as feeders in KSR/DMEM medium plus PD0325901 and LIF (K/DL) and cultured for 7 days to form outgrowths. Emerging ICM outgrowths were directly picked and placed onto feeder cells in serum/LIF (S/L) medium to establish stable ESC lines. Established ESC lines were genotyped by *Sry* gene to determine the sex. Female ESC lines were also maintained in serum/LIF (S/L) medium and passaged by dissociating cells with 0.25% TE (Invitrogen) every 2–3 days and re-plated onto feeder cells. K/DL medium contains knockout DMEM (Invitrogen) supplemented with 20% Knockout serum replacement (KSR, Invitrogen), 1 mmol/L l-glutamine, 1% nonessential amino acid stock, 50 units/mL penicillin and 50 μg/mL streptomycin (Invitrogen), 0.1 mmol/L β-mercaptoethanol (Invitrogen), 1 μmol/L PD0325901 (Miltenyi) and 1000 IU/mL mouse LIF (mLIF, Millipore). S/L medium (ESC culture medium) contains knockout DMEM supplemented with 15% FBS (ES quality, Hyclone), 1 mmol/L l-glutamine, 1% nonessential amino acid stock, 50 units/mL penicillin and 50 μg/mL streptomycin, 0.1 mmol/L β-mercaptoethanol, and 1000 IU/mL mLIF.

### Karyotype analysis

Metaphase chromosomes were prepared by exposing cultured cells to nocodazole (0.3 μg/mL) for 3 h, followed by hypotonic treatment with 75 mmol/L KCl solution, fixed with methanol:glacial acetic acid (3:1) and spread onto clean slides under humidified cold environment. About 30 separate metaphase spreads were examined for each culture.

### Induction of PGC-like cells (PGCLCs)

Induction of PGCLCs from pESCs/ESCs was based on the method described previously (Hayashi et al. 2012; Hayashi and Saitou 2013). ESCs and pESCs were maintained in t2iL medium (in 2iL medium, the concentration of PD0325901 was reduced from 1 μmol/L to 0.4 μmol/L) for 3–5 passages. EpiLCs were induced by plating 1.0 × 10^5^ pESCs/ESCs on the wells of a 12-well plate coated with human plasma fibronectin (FN, 16.7 μg/mL, Millipore) in N2B27 medium containing activin A (20 ng/mL, PeproTech), bFGF (12 ng/mL), and 1% KSR for 46 h. The medium was changed every day. The PGCLCs were induced for 4 days under floating conditions by plating 3.0 × 10^3^ EpiLCs in the wells of a low-cell-binding U-bottom 96-well Lipidure-Coat plate (Corning) in GK15 medium (GK15 medium contains GMEM (Invitrogen) added with 15% KSR, 100 μmol/L NEAA, 1 mmol/L sodium pyruvate, 1 mmol/L l-glutamine, 1% nonessential amino acid stock, 50 units/mL penicillin and 50 μg/mL streptomycin, 0.1 mmol/L β-mercaptoethanol) in the presence of the cytokines BMP4 (500 ng/mL, PeproTech), 1000 IU/mL mLIF, SCF (100 ng/mL, PeproTech) and EGF (50 ng/mL, PeproTech). Integrin-β3- (CD61) and SSEA1-double positive PGCLCs were sorted by flow cytometer. Dissociated cells were incubated with anti-integrin-β3 (CD61) antibody and anti-SSEA1 antibody conjugated with PE (Phycoerythrin) and APC (Allophycocyanin), respectively. After being washed in PBS supplemented with 0.1% BSA, the cells were sorted and analyzed on a flow cytometer (Aria III; BD Biosciences).

### Induction of meiosis and folliculogenesis

Induction of meiosis and folliculogenesis was achieved by aggregation of PGCLCs with E12.5 gonadal somatic cells followed by transplantation of the aggregates into kidney capsules to generate reconstituted ovaries (Qing et al. 2008). To obtain E12.5 gonad somatic cells *in vivo*, female E12.5 gonads were collected from E12.5 embryos obtained by intercrosses of albino ICR mice. The mesonephros were surgically separated from the gonads using insulin syringe. Gonads were dissociated with 0.05% TE by incubation at 37 °C for 10 min, washed with MEF medium, and collected by centrifugation. Large clumps of cells were removed using a cell strainer (BD Biosciences). Endogenous PGCs in the dissociated gonadal cells were removed or collected by magnetic cell sorting using anti-SSEA1 antibody conjugated with magnetic beads (Miltenyi, see below). The sorted gonadal somatic cells and FACS-sorted PGCLCs were plated in the wells of a low-cell-binding U-bottom 96-well Lipidure-Coat plate in MF10 medium (MF10 medium contains M199 (Sigma) added with 10% FBS, 1 mmol/L l-glutamine, 50 units/mL penicillin and 50 μg/mL streptomycin, 50 μg/mL Vitamin C (Vc, Sigma) and 10 μmol/L Rocki (Y27632, Rock inhibitor) (Tian et al. 2019). About 20,000 PGCLCs and 100,000 gonadal somatic cells were mixed per aggregate. For negative control (pseudo-rOvary), 100,000 E12.5 gonadal somatic cells only without PGCs were aggregated in MF10 medium supplemented with 50 μg/mL Vc and 10 μmol/L Rocki.

### Magnetic activated cell sorting

Magnetic activated cell sorting **(**MACS) was performed according to the manufacturer’s instructions (Miltenyi). Briefly, dissociated gonadal cells were incubated with anti-SSEA1 antibody conjugated with magnetic beads. Cell suspensions were washed in PBS supplemented with 0.5% BSA and 2 mmol/L EDTA and applied to an MS column (Miltenyi) to remove SSEA1-positive PGCs. Gonadal somatic cells were collected in the flow-through portions. To verify that SSEA1 negative cells did not contain PGCs, SSEA1/CD61 double staining was used to detect PGCs by FACS. More than 99.99% cells did not express SSEA1 and CD61, showing the purity of embryonic gonadal somatic cells.

### Isolation of E9.5 and E12.5 PGCs

Embryonic gonads were dissected from E9.5 and E12.5 female embryos. Gonads were dissociated by 0.25% TE (Invitrogen), and filtered by 70 μm cell Filter, followed by sorting of PGCs by flow cytometer using anti-integrin-β3 (CD61) antibody and anti-SSEA1 antibody or MACS using anti-SSEA1 antibody conjugated with magnetic beads.

### Kidney capsule transplantation

Kidney capsule transplantation was performed based on the methods described (Qing et al. 2008). Briefly, one or two aggregates were implanted in the “pocket” which was made between the kidney capsule and kidney tissue of a bilaterally ovariectomized recipient mouse. Transplantation procedure was completed in 5 min for each mouse. Meiosis and folliculogenesis were achieved in the reconstituted ovaries 26–28 days following transplantation of the aggregates.

### Follicle count

The aggregate-formed reconstituted ovaries were carefully retrieved and subsequently dehydrated with graded alcohols, cleared in xylene, and embedded in paraffin wax. The serial sections (5 μm) from each reconstituted ovary were aligned in order on glass microscope slides, stained with H&E and analyzed for the number of follicles at various developmental stages in every fifth section with random start in the first five sections. The total number of follicles per reconstituted ovary was calculated by combining the counts of every fifth section throughout the whole reconstituted ovary, based on method described (Liu et al. 2013). The follicles were categorized into, secondary and antral or mature accordingly. Secondary follicles were characterized as having more than one layer of GCs with no visible antrum. Antral or mature follicles possessed small areas of follicular fluid (antrum) or a single large antral space. Only those follicles containing an oocyte with a clearly visible nucleus were scored.

### *In vitro* maturation and *in vitro* fertilization

The reconstituted ovaries were dissected from the recipient mouse kidney capsule, and fully-grown GV oocytes were collected under a microscope by pricking follicles using insulin syringe in *in vitro* maturation (IVM) medium. Oocytes were matured *in vitro* by culture in IVM medium for 17–18 h at 37 °C (Eppig et al. 2009). IVM medium contained α-MEM (Invitrogen) added with 5% FBS, 0.24 mmol/L sodium pyruvate, 1 IU/mL PMSG and 1.5 IU/mL hCG. With this IVM method, 80% of maturation rate was routinely obtained for oocytes collected *in vivo*.

For *in vitro* fertilization (IVF), spermatozoa were collected from the cauda epididymis of albino ICR males, capacitated by incubation for 2 h in HTF (Origio), and then incubated with the matured oocytes for 6 h. The zygotes were transferred into human G-1 plus medium (Vitrolife). Embryos that reached the 2-cell stage after 24 h culture were transferred into the oviducts of E0.5 pseudo pregnant mice (albino Kunming mice) and newborns were normally delivered on E19.5. Pups were identified initially by coat color. The contribution of pESCs and ESCs of pups was confirmed by standard DNA microsatellite genotyping analysis using D8Mit94 and D12Mit136 primers (Table S4).

### Test of developmental pluripotency by generation of chimeras

Approximately 10–15 pESCs or ESCs were injected into 4–8-cell embryos of albino not Balb/c as hosts using a Piezo injector as described (Huang et al. 2008). Injected embryos were cultured overnight in KSOM_AA_ medium. Blastocysts were transferred into uterine horns of E2.5 surrogate albino Kunming mice. Pregnant females delivered pups naturally at about E19.5. Pups were identified initially by coat color. Chimeras were mated with albino ICR mice to further examine their germline transmission competence.

### Immunofluorescence microscopy

Cells were washed twice in PBS, fixed in freshly prepared 3.7% paraformaldehyde for 30 min on ice, washed once in PBS and permeabilized in 0.1% Triton X-100 in blocking solution (3% goat serum plus 0.1% BSA in PBS) for 30 min at room temperature, then washed three times in PBS, and left in blocking solution for 2 h. Cells were incubated overnight at 4 °C with primary antibodies, anti-Oct4 and anti-Nanog prepared in blocking solution, washed three times for 15 min each with blocking solution, followed by incubation for 2 h with secondary antibodies at room temperature. Alexa Fluor® 594 goat anti-mouse IgG (H+L) against anti-Oct4 or Alexa Fluor® 594 goat anti-rabbit IgG (H+L) against anti-Nanog diluted in 1:200 with blocking solution was used. Samples were washed, and counterstained with 0.5 μg/mL DAPI (Roche) in Vectashield (Vector) mounting medium. Fluorescence was detected and imaged using Axio-Imager Z2 Fluorescence Microscope (Carl Zeiss).

### Fluorescence microscopy of reconstituted ovarian sections

Briefly, after being deparaffinized, rehydration and wash in 0.01 mol/L PBS (pH 7.2), sections were subjected to high pressure antigen recovery sequentially in 0.01 mol/L citrate buffer (pH 6.0) for 3 min, so that the autofluorescence of Actin-GFP was quenched. The sections were incubated with blocking solution (3% BSA in PBS) for 2 h at room temperature, and then with the diluted primary antibodies overnight at 4 °C. The primary antibodies included anti-SCP3, GFP, Vasa, Dazl, Foxl2, or Gata4 prepared in blocking solution. Blocking solution without the primary antibody served as negative control. After washing with PBS three times, sections were incubated with appropriate secondary antibodies, AlexaFluor® 594 Donkey anti-Goat IgG (Foxl2), AlexaFluor® 594 Goat Anti-Mouse IgG (Gata4), AlexaFluor® 488 Donkey anti-Mouse IgG (GFP), AlexaFluor® 488 Donkey anti-Rabbit IgG (SCP3, Vasa, and Dazl) or AlexaFluor® 594 Goat Anti-Rabbit IgG (Dazl in the sections of day 4 reconstituted ovaries). Nuclei were stained with 0.5 μg/mL DAPI in Vectashield mounting medium. Fluorescence was detected and imaged using Axio-Imager Z2 Fluorescence Microscope (Carl Zeiss).

### Immunostaining and fluorescence microscopy of meiocyte spreads

Surface spreading of meiocytes was prepared by a drying-down technique and stained for synaptonemal complexes (Liu et al. 2004). PGCLC-aggregates were collected, minced with two forceps and dissociated by pipetting in 0.05% TE. After incubation for 7 min at 37 °C, cell suspensions were mixed with an equal volume of FBS, centrifuged for 5 min and resuspended in 100 mmol/L sucrose. The cell suspension was spread onto glass slide by dipping onto a thin layer of fixative (1% paraformaldehyde, 0.15% Triton X-100 and 3 mmol/L dithiothreitol, pH 9.2). The glass slides were maintained overnight in a humidified box at 4 °C. The slides were washed in water containing 0.4% Photo-flow (Kodak), and completely dried at room temperature. Dried slides were washed with 0.1% Triton X-100/PBS (PBST) for 10 min and incubated with antibody dilution buffer (ADB) (3% BSA and 2% goat serum in PBST) for 1 h at room temperature. Spreads were then incubated with anti-SCP1, SCP3 or MLH1 antibody in ADB at 4 °C overnight, washed three times, then incubated with appropriate secondary antibodies, AlexaFluor® 488 Goat anti-Rabbit IgG or AlexaFluor® 594 Goat anti-Rabbit IgG (SCP3), FITC Goat anti-Mouse IgG (H+L) (MLH1) and AlexaFluor® 594 Goat anti-Mouse IgG (SCP1). After washing, samples were stained with DAPI, washed and mounted in Vectashield mounting medium (Vector Laboratories). Immunofluorescence was detected using Axio-Imager Z2 Fluorescence Microscope. MLH1 foci were counted as described (Liu et al. 2004). Autofluorescence of Actin-GFP was not observed in these spreads.

### Hormone assays

Serum follicle-stimulating hormone (FSH), estradiol (E2) and anti-Müllerian hormone (AMH) levels were assayed by ELISA kit (CK-E20381, CK-E20419 and CK-E90200, Hangzhou EastBiopharm CO., LTD). Quality control serum, sterilized distilled water, and five series diluted standard samples for a standard curve were tested for each serum sample. The intra- and inter-assay coefficients of variability for AMH, FSH, and E2 were below 8% and 12%.

### Gene expression analysis by real-time qPCR

Total RNA was purified using RNA mini kit (QIAGEN), treated with DNase I (QIAGEN), and the cDNA was generated from 2 μg RNA using Oligo (dT)18 primer (Takara) and M-MLV Reverse Transcriptase (Invitrogen). Real-Time qPCR reactions were set up in duplicate with the FS Universal SYBR Green Master (Roche) and carried out on an iCycler MyiQ2 Detection System (Bio-Rad). All reactions were carried out by amplifying target genes and internal control in the same plate. Each sample was repeated two times and normalized using *Gapdh* as the internal control. The amplification was performed for primary denaturation at 95 °C for 10 min, then 40 cycles of denaturation at 95 °C for 15 s, annealing and elongation at 58 °C for 1 min, and the last cycle under 55–95 °C for dissociation curve. Relative quantitative evaluation of target gene was determined by comparing the threshold cycles. Primers (Table S5) were confirmed for their specificity with dissociation curves.

### Combined bisulfite restriction analysis (COBRA)

COBRA analysis was performed based on the methods as described (Hikabe et al. 2016). Genomic DNA was extracted using DNeasy Tissue Kit (QIAGEN) according to the manufacturer’s instructions. Bisulfite treatment of DNA was performed with the EpiTect Bisulfite Kit (QIAGEN). Bisulfite converted DNA was amplified by PCR, using HS Taq DNA Polymerase (QIAGEN) and primers (Table S6). Thermal cycling was carried out with a 10 min denaturation step at 94 °C, followed by 35 three-step cycles (30 s at 94 °C, 30 s at 55–58 °C and 30 s at 72 °C) and final incubation at 72 °C for 10 min. PCR products were recovered from stained gels (Gel Extraction Kit, Transgene). The amplified DNA was digested with the following restriction enzymes (NEB): AciI for *Snrpn* and *Igf2r*; PvuI-HF for *Dlk1*/*Glt2*; HpyCH4IV for *H19*/*Igf2r* and *Mest1*. The digested samples were resolved through electrophoresis in 1.5% agarose gels and illuminated by GelRed (BioSharp).

### Library preparation and RNA-sequencing

1000 Cells per sample were resuspended in PBS with 0.1% BSA and transferred to the bottom of a PCR tube consisting of 3 μL lysis buffer, and cDNA was synthesized in the tube containing mRNA, based on Smart-seq2 protocol (Picelli et al. 2014), and the libraries were prepared using TruePrep DNA Library Prep Kit V2 for Illumina® (TD503-02, Vazyme Biotech) according to the manual instruction. Samples were barcoded and multiplex sequenced with a 150-bp paired-end sequencing strategy on an Illumina Hiseq platform.

### Library preparation and reduced representation bisulfite sequencing (RRBS)

Cells were collected and isolated their genomic DNA by QIAamp DNA Micro Kit (QIAGEN). DNA purity was checked using the NanoPhotometer® spectrophotometer (IMPLEN, CA, USA) and DNA concentration was measured using Qubit® DNA Assay Kit in Qubit® 2.0 Flurometer (Life Technologies, CA, USA). A total amount of 1.5 μg DNA spiked with moderate lambda DNA was handled by MspI, followed by end repair and adenylation. Cytosine-methylated barcodes were ligated to sonicated DNA following manufacturer’s instructions. The DNA fragments were treated twice with bisulfite using EZ DNA Methylation-GoldTM Kit (Zymo Research), and the resulting single-strand DNA fragments were PCR amplified using KAPA HiFi HotStart Uracil and ReadyMix (2X). Library concentration was quantified by Qubit® 2.0 Flurometer and quantitative PCR, and the insert size was assayed on Agilent Bioanalyzer 2100 system. The library preparations were sequenced on an Illumina Novaseq platform and 125 bp/150 bp paired-end reads generated. Image analysis and base calling were performed with Illumina CASAVA pipeline.

Bismark software (version 0.16.3) was used to perform alignments of bisulfite-treated reads to a reference genome. The reference genome was firstly transformed into bisulfite-converted version (C-to-T and G-to-A converted) and then indexed using bowtie2. Sequence reads were also transformed into fully bisulfite-converted versions (C-to-T and G-to-A converted) before they were aligned to similarly converted versions of the genome in a directional manner. Sequence reads that produced a unique best alignment from the two alignment processes (original top and bottom strand) were then compared to the normal genomic sequence and the methylation state of all cytosine positions in the read was inferred. The same reads that were aligned to the same regions of genome were regarded as duplicates. The sequencing depth and coverage were summarized using duplicated reads. Results of methylation extractor were transformed into bigWig format for visualization using IGV browser. Sodium bisulfite non-conversion rate was calculated as the percentage of cytosine sequenced at cytosine reference positions in the genome.

### Bioinformatics analysis

For RNA-seq, clean reads were mapped to the mouse reference mm10 reference genome using Hisat2. Reads were assigned and counted to genes using the Feature-counts. The resulting matrix of read counts was then loaded into RStudio (R version 3.4.2), and DESeq2 used to identify differentially expressed genes. Functional enrichment (GO annotation or KEGG) of gene sets with different expression patterns was performed using clusterProfiler. The heatmaps were drawn by the function “pheatmap” of R packages and correlation coefficients calculated by the function “cor” in R. Scatter plots were generated using the “ggplot2” package to graphically reveal genes that differ significantly between two samples. The *P* values were adjusted using the Benjamin and Hochberg method (Hochberg and Benjamini 1990). Corrected *P*-value of 0.05 and two-fold changes were set as the threshold for significantly differential gene expression. The PGC marker gene list was obtained based on previous published RNA-seq data (Miyauchi et al. 2017). Calculated Zscore, log_10_(TPM), log_10_(TPM+1) or log_2_(TPM+1) of selected genes was used for heatmap.

For RRBS, we modeled the sum Mc of methylated counts as a binomial (Bin) random variable with methylation rate r to identify the methylation site. In order to calculate the methylation level of the sequence, we divided the sequence into multiple bins, with bin size at 10 kb. The sum of methylated and unmethylated read counts in each bin were calculated. Methylation level (ML) for each bin or C site shows the fraction of methylated C, and is defined as: ML(C) = reads(mC)/[(reads(mC) + reads(C)). Calculated ML was further corrected with the bisulfite non-conversion rate by: ML (corrected) = (ML − r)/(1 − r). Analysis of fraction of CpGs in global methylation levels and methylation heatmap for various genomic features were based on published methods (Seisenberger et al. 2012; Wang et al. 2014). Analysis of DNA methylation levels of maternal and paternal imprints was based on the published gene list (Xie et al. 2012; Li et al. 2016).

### Quantification and statistical analysis

Statistics were analyzed by using the StatView software from SAS Institute, Inc. (Cary, NC). Data were analyzed using two-tailed unpaired Student’s t test to compare two groups or ANOVA to compare more than two groups and expressed as mean ± SEM. p values less than 0.05 were considered significant (*P < 0.05, **P < 0.01 or ***P < 0.001). The exact values of “*n*” used are described in the corresponding figure legends. “*n*” refers to the number of biological replicates and includes either number of mice or replicates of cell studies. FACS data was analyzed by FlowJo. Graphs were generated using GraphPad Prism or R package ggplot2 or other R packages.

## ABBREVIATIONS

AMH, anti-Müllerian hormone; DMR, differentially methylated region; E2, estradiol; EB, embryoid body; EpiLCs, epiblast-like cells; ESC, embryonic stem cell; FACS, fluorescence-activated cell sorting; FSH, follicle-stimulating hormone; GFP, green fluorescence protein; GO, gene ontology; GV, germinal vesicle; H&E, hematoxylin and eosin stain; ICSI, intracytoplasmic sperm injection; iPSC, induced pluripotent stem cell; IVF, * in vitro* fertilization; IVM, * in vitro* maturation; NOD-SCID mice, non-obese diabetic-server combined immune-deficiency mice; OE, bilaterally oophorectomized; PCA, principal components analysis; pESC, parthenogenetic embryonic stem cell; PGC, primordial germ cell; PGCLC, primordial germ cell-like cell; RA, retinoic acid; RRBS, reduced representation bisulfite sequencing; SC, synaptonemal complex.

## Supplementary Information

Below is the link to the electronic supplementary material.

Supplementary file1 (PDF 1048 kb)

## DECLARATIONS

### Data Availability

The accession number for RNA sequencing data reported in this paper is NCBI GEO: GSE141103 and GSE154144, and RRBS data is NCBI GEO: GSE153979. Information on oligonucleotide sequences can be found in the Tables S4, S5 and S6. Other relevant data and reagents that support the findings of this study are available from the corresponding author on reasonable request. Additional data related to this paper may be requested from the authors.

### Code availability

All the custom codes written in R is available from the corresponding authors upon reasonable request.
