# Supplementary material for: Generation of developmentally competent oocytes and fertile mice from parthenogenetic embryonic stem cells
Source: Protein Cell. 2021 Nov 30;12(12):947–64. doi: 10.1007/s13238-021-00865-4 (PMC8674391; doi:10.1007/s13238-021-00865-4)
Supplement: Supplementary file 1 — Supplementary file1 (PDF 1048 kb) [file 13238_2021_865_MOESM1_ESM.pdf]

Supplementary Materials for

Generation of developmentally competent oocytes and fertile mice from parthenogenetic embryonic stem cells

Chenglei Tian, Linlin Liu, Ming Zeng, Xiaoyan Sheng, Dai Heng, Lingling Wang, Xiaoying Ye, David L. Keefe, Lin Liu

The PDF file includes:

Figs. S1 to S8

tables S1 to S6

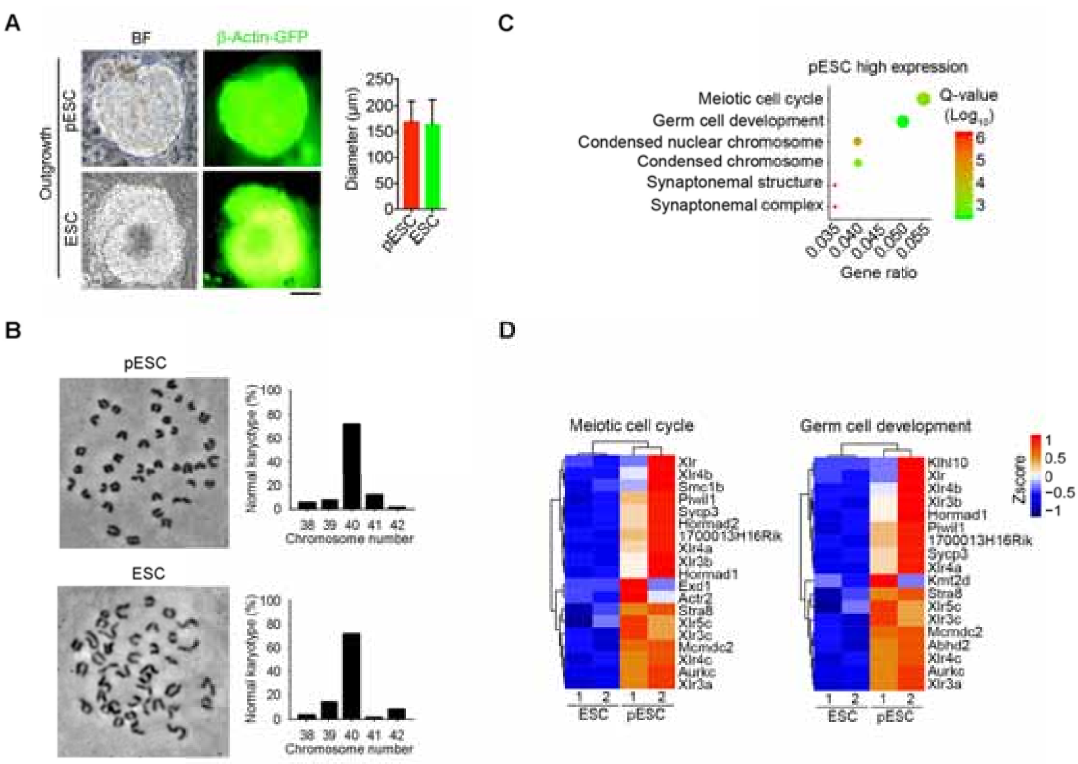

Fig. S1. Derivation of pESCs and ESCs. (**A**) Morphology and Actin-GFP expression of pESC- and ESC-outgrowths derived from blastocysts (left) and size in diameter of the outgrowth (right). Scale bar = 100 μm. (**B**) Karyotype analysis of pESCs and ESCs. (**C**) Enriched GO items of upregulated genes in pESCs compared with ESCs. Two-fold changes ($p < 0.05$) are set as threshold. (**D**) Typical upregulated genes of pESCs in meiotic cell cycle (left) and germ cell development (right) revealed by GO analysis. All RNA-seq analysis represents two biological replicates.

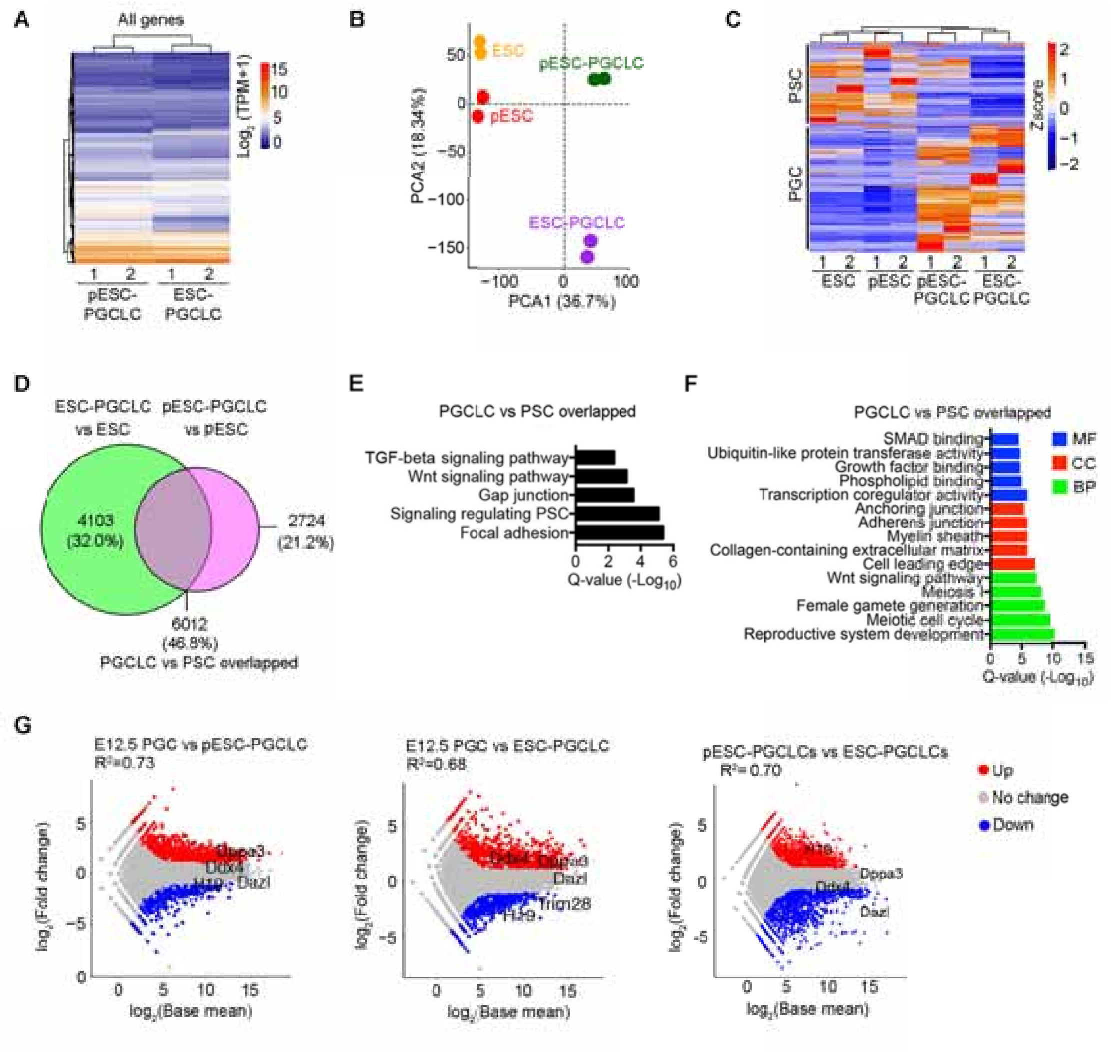

Fig. S2. Gene expression profile of pESC- and ESC-PGCLCs by RNA-seq. (**A**) Heatmap showing similar global expression profile between pESC-PGCLCs and ESC-PGCLCs. (**B**) PCA analysis of pESCs, ESCs, pESC-PGCLCs and ESC-PGCLCs by RNA-seq. (**C**) Heatmap displaying expression profile of PSC and PGC genes in pESCs, ESCs, pESC-PGCLCs and ESC-PGCLCs. Color key from red to blue represents the relative gene expression level from high to low. (**D**) Venn diagram displaying differentially expressed genes between pESC-PGCLCs and pESCs or between ESC-PGCLCs and ESCs. Two-fold changes ($p < 0.05$) are set as threshold for each group. (**E**) KEGG analysis of overlapped genes in (**D**) ($p < 0.05$). PSCs, ESCs or pESCs. (**F**) GO analysis of overlapped genes in (**D**) ($p < 0.05$). MF, Molecular Function; CC, Cellular Component; BP, Biological Process. **(G)** Scatter-plots showing comparison of genome-wide transcription of pESC-PGCLCs, ESC-PGCLCs and E12.5 PGCs. R^2, squared of Pearson's correlation coefficient. Two-fold changes ($p < 0.05$) are set as threshold for each group. All RNA-seq analysis represents two biological replicates.

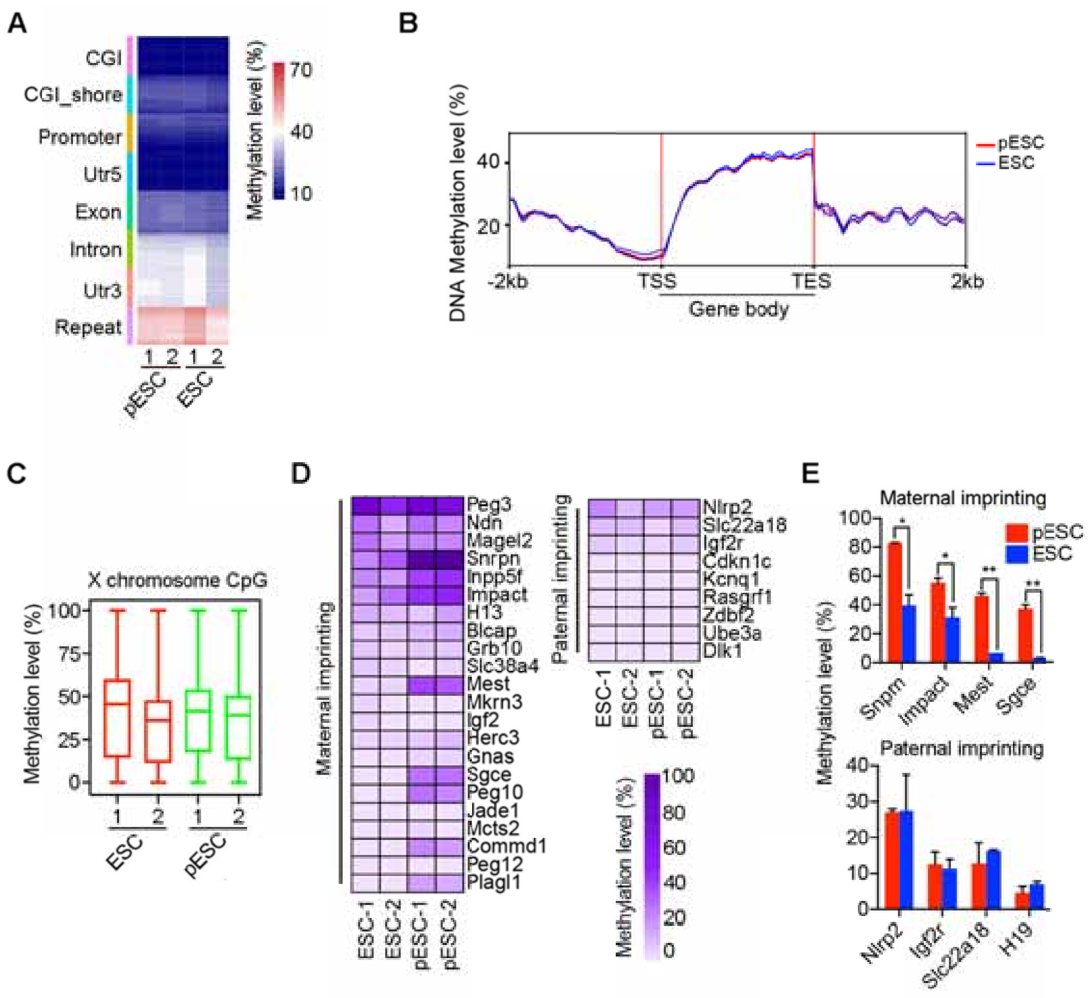

Fig. S3. DNA methylation of ESCs and pESCs. (**A**) Heatmap showing genome-wide DNA methylation levels at different gene regions of ESCs and pESCs. (**B**) Genome-wide DNA methylation level in gene body regions including up- and down-stream 2kb of gene body. (**C**) Box plot showing global DNA methylation levels in X chromosomes. (**D**) Methylation levels of maternal and paternal imprinting genes in pESCs and ESCs. Methylation counts are provided in table S1. (**E**) Methylation levels of typical maternal and paternal imprinting genes in pESCs and ESCs. All RRBS analysis represents two biological replicates.

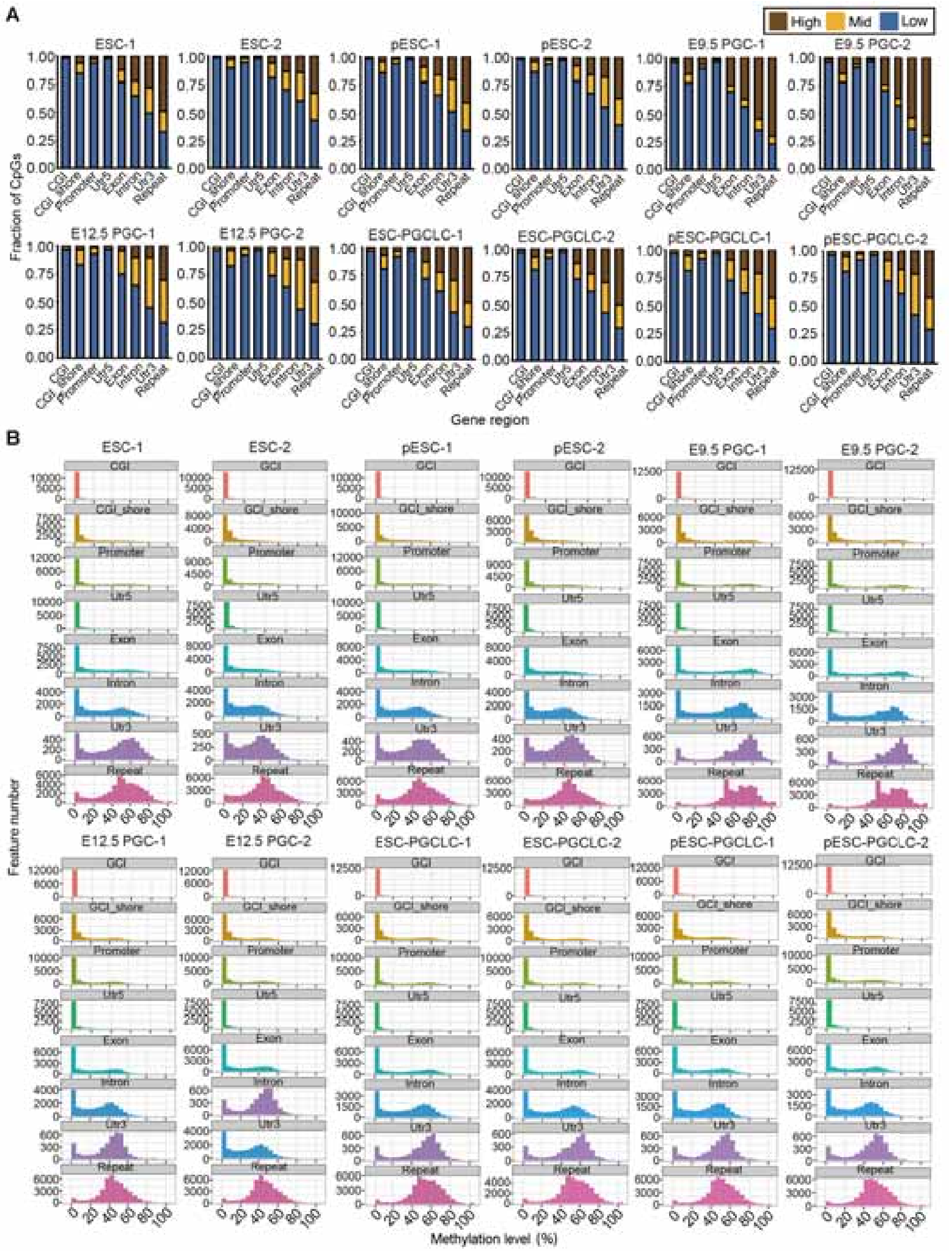

Fig. S4. Global DNA methylation profile of ESCs and pESCs, pESC- or ESC-PGCLCs, E9.5 PGCs and E12.5 PGCs by RRBS. (**A**) Summary of high, middle (Mid) and low fraction of methylated CpGs at the different gene regions in pESCs, ESCs, pESC-PGCLCs, ESC-PGCLCs, E9.5 PGCs and E12.5 PGCs. High, 60-100%; middle, 30-60%; low, 0-30%. (**B**) Feature number and methylation levels at GCI, GCI_shore, Promoter, Utr5, Exon, Intron, Utr3 and Repeat in pESCs, ESCs, pESC-PGCLCs, ESC-PGCLCs, E9.5 PGCs and E12.5 PGCs. It is to note that the methylome experiments for pESCs in comparison with ESCs, and for comparison of PGCLCs from pESCs/ESCs

with *in vivo* PGCs were performed at different time. Here we did not intend to directly compare methylome of PGCLCs with that of their progenitors ESCs/pESCs.

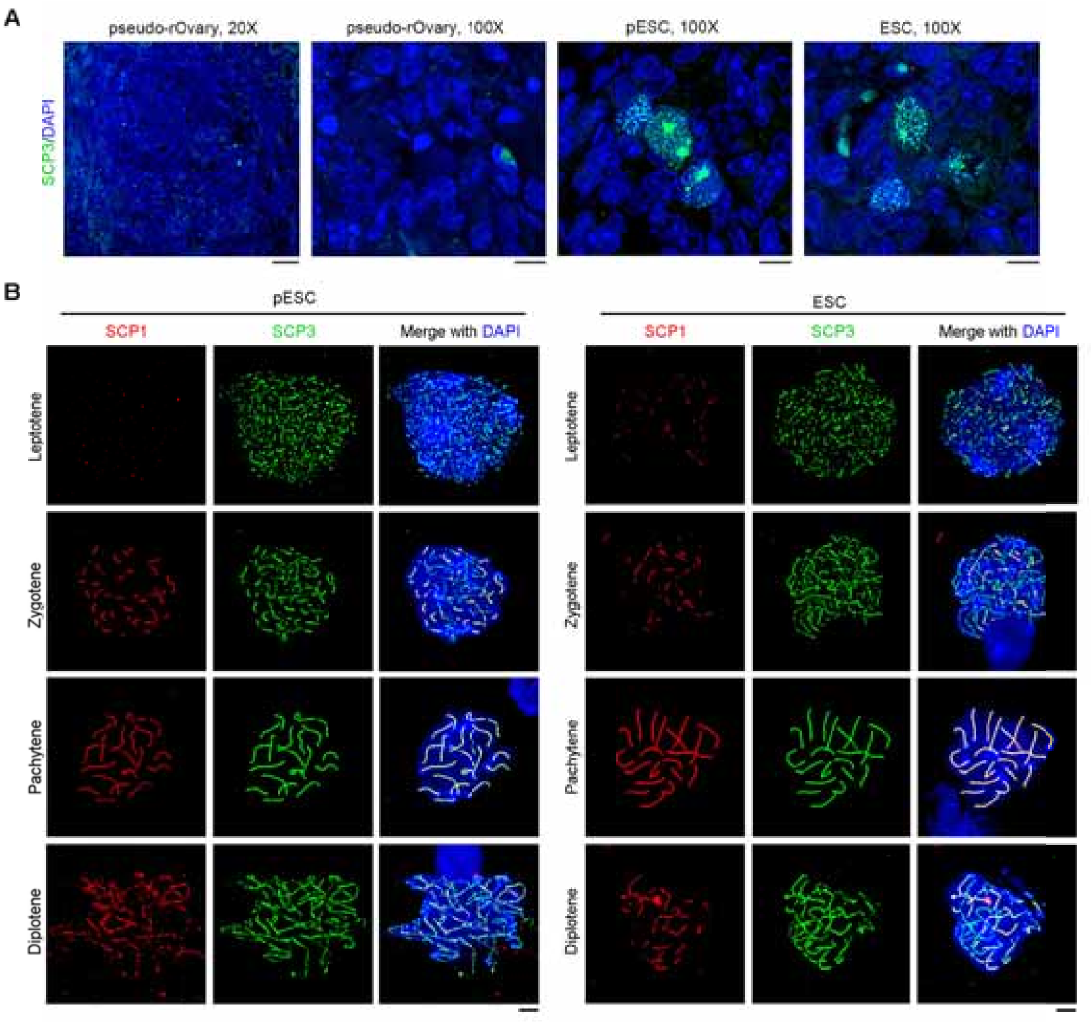

Fig. S5. Meiosis in reconstituted ovaries derived from pESC- or ESC-PGCLCs. (**A**) Pachytene spread is not observed in pseudo-reconstituted ovaries (rOvaries) but observed in pESC- and ESC-reconstituted ovaries by immunofluorescence of the paraffin sections. The reconstituted ovary samples were collected 5 days following transplantation of the aggregates. 20X, scale bar = 100 μm; 100X, scale bar = 10 μm. (**B**) Immunofluorescence of spread of pESC- and ESC- reconstituted ovaries formed from the aggregates 5 days following transplantation, revealing SCP1 (red) and SCP3 (green) lateral filaments in meiocytes at leptotene, zygotene, pachytene, and diplotene stages of prophase I. Scale bar = 5 μm.

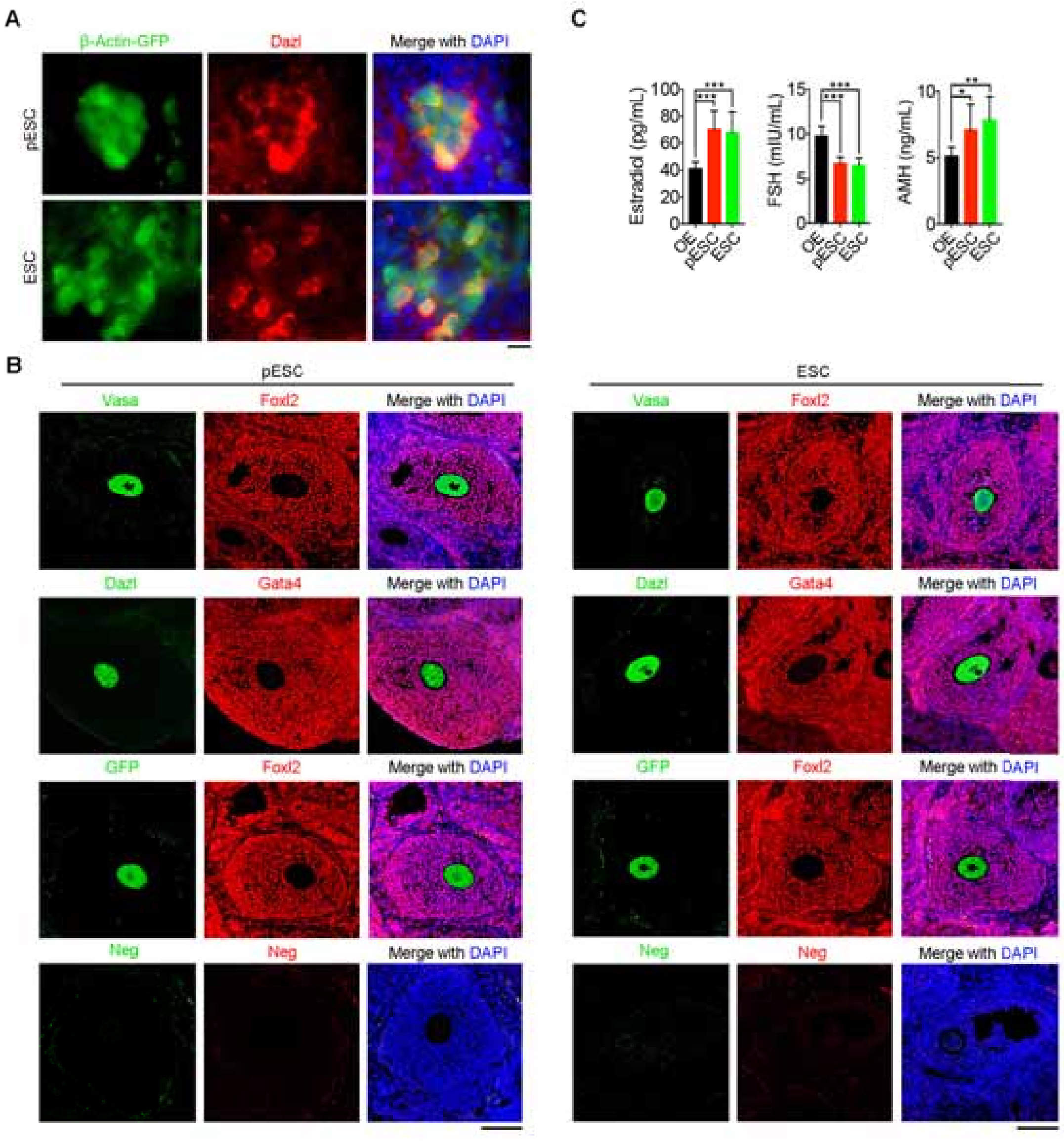

Fig. S6. Folliculogenesis in reconstituted ovaries from pESC- or ESC-PGCLCs. (**A**) Representative immunofluorescence of Actin-GFP and Dazl expression in pESC- or ESC-reconstituted ovaries 4 days after transplantation of the aggregates to the kidney capsule. Scale bars = 10 µm. (**B**) Representative immunofluorescence of Actin-GFP and typical germ cell markers Vasa and Dazl expressed in pESC- or ESC-reconstituted ovaries 28 days after transplantation of the aggregates to the kidney capsule. Immunostaining without primary antibody served as negative control (Neg). Scale bar = 100 µm. (**C**) Bilateral ovariectomy (OE) mice transplanted with the reconstituted ovaries generated from pESCs or ESCs exhibit elevated levels in serum of E2 and AMH and reduced FSH by ELISA assay, compared with OE mice without transplants (n = 8). Mean ± SEM. *$p < 0.05$, **$p < 0.01$, ***$p < 0.001$. Despite variations among different batches, the hormone levels of the recipients receiving transplants in the present study generally

are comparable to those of normal mice reported by other groups (Kevenaar et al. Endocrinology 147:3228–3234 (2006); Niringiyumukiza et al. Biomedicine & Pharmacotherapy 116:108963 (2019)).

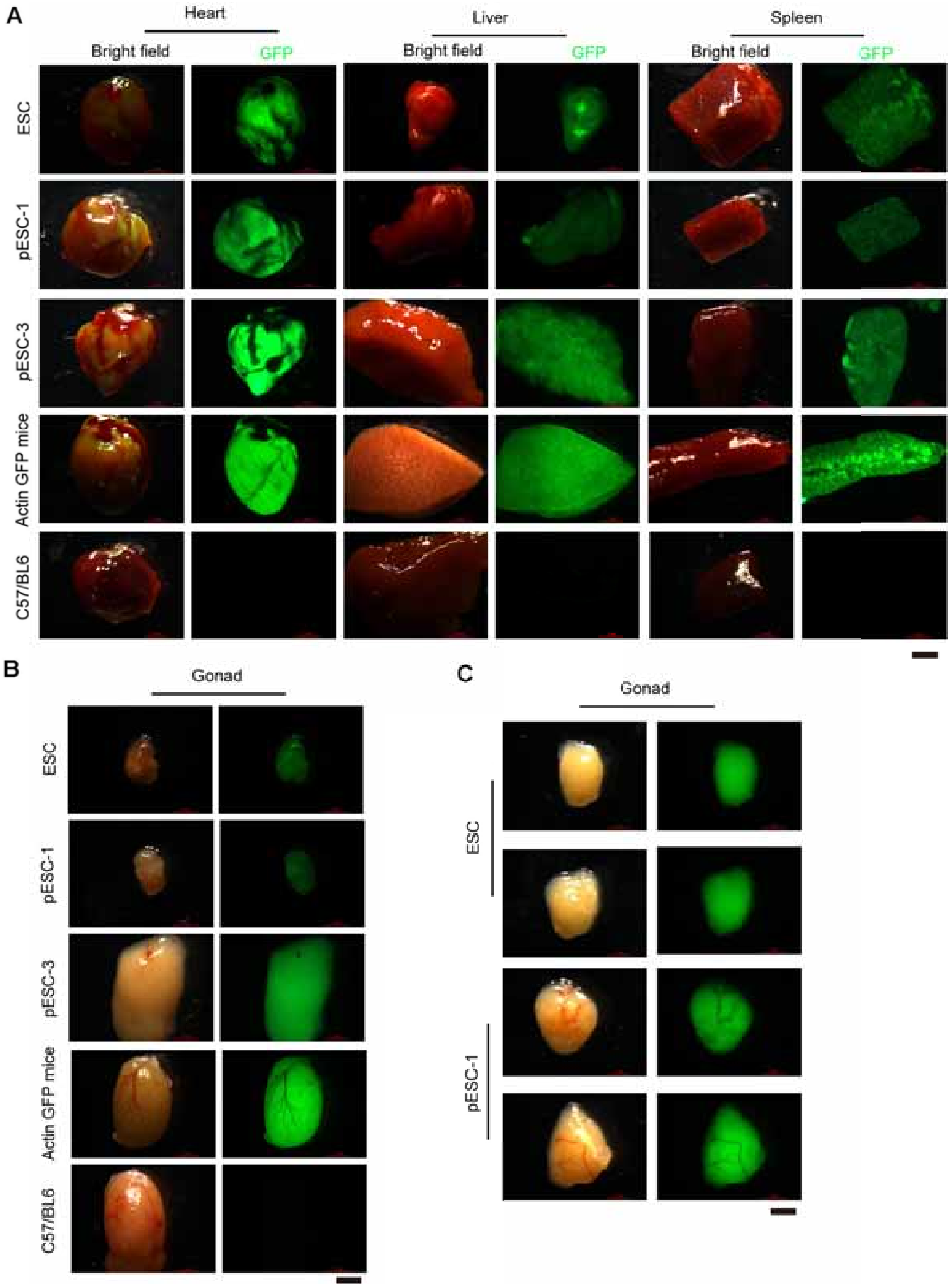

Fig. S7. GFP fluorescence in the tissues of mice produced from pESCs or ESCs. (A,B) Representative tissue samples of mice from pESCs or ESCs (AKJ3) such as heart, liver, spleen and gonad exhibit GFP fluorescence imaged by stereo fluorescence microscopy. Tissue samples were collected from ESC- and pESC-PGCLC derived mice and stored

frozen at -80 °C. (**C**) Representative gonad collected from the offspring derived from ESC- and pESC-PGCLC derived mice imaged by stereo fluorescence microscopy. Gonads (ovaries, small; testis, larger) were collected and stored frozen at -80 °C. All images were taken at the same time by the same exposure time. Tissue samples collected from normal C57BL/6 mice without carrying GFP served as negative control. Fresh samples collected from Actin-GFP mice served as positive control for GFP fluorescence. Scale bar = 2 mm.

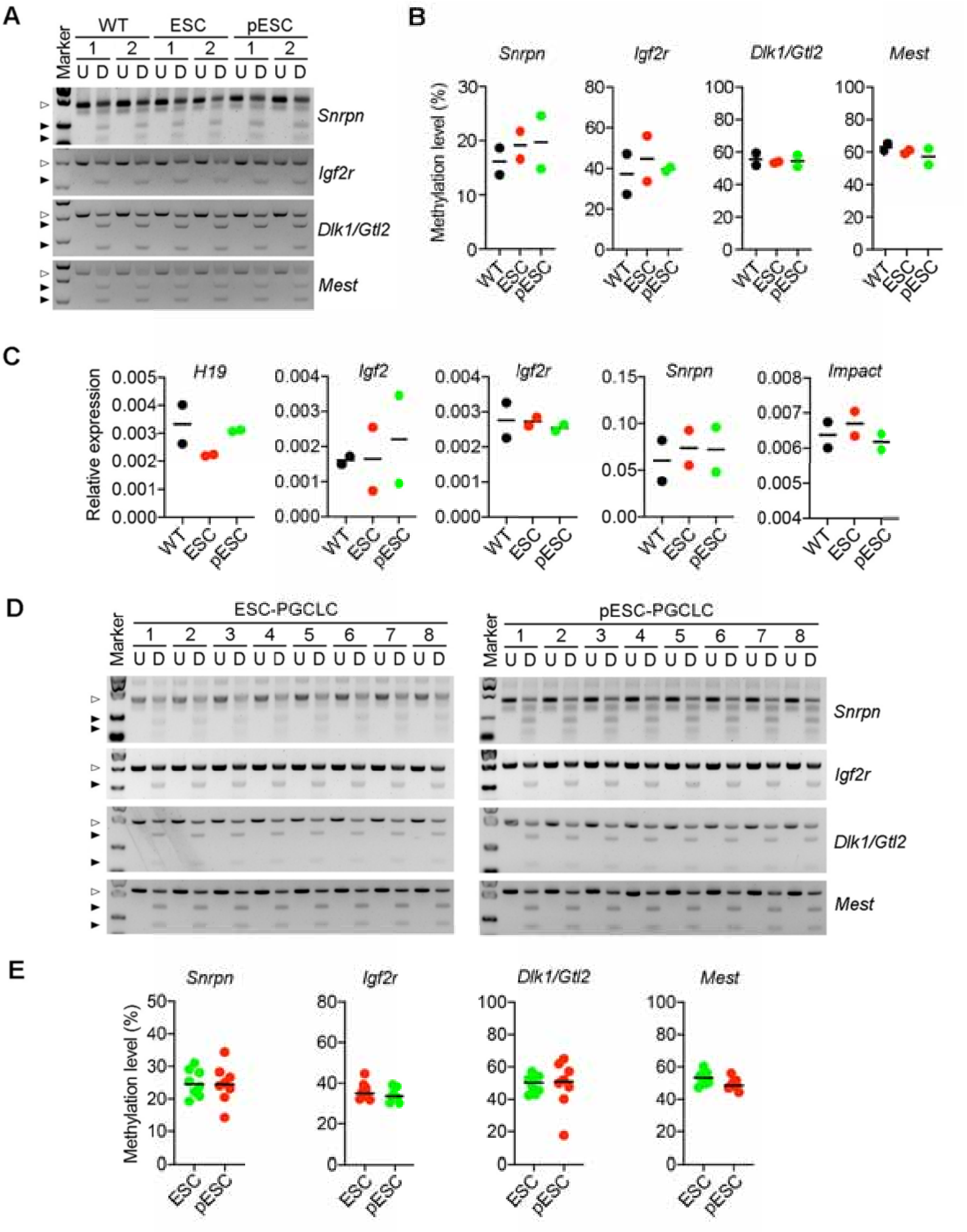

Fig. S8. Imprinting of PGCLCs-derived mice. (**A**) Combined bisulfite restriction analysis (COBRA) of typical imprints (*Snrpn*, *Igf2r*, *Dlk1/Gtl2* and *Mest*) of tail tissue from ESC- and pESC-PGCLC derived mice (n = 2). Wild-type (WT) mice from normal breeding at the same background served as control. PCR products were either digested (D) or undigested (U) with the respective enzyme. The digested and undigested fragments are indicated by black and white triangles, respectively. (**B**) Methylation level analysis of

COBRA by Image J. (**C**) Expression of typical imprinting genes (*H19*, *Igf2*, *Igf2r*, *Snrpn* and *Impact*) of tail tissue from mice derived from ESC-PGCLCs and pESC-PGCLCs. Same background WT mice served as control. (**D**) COBRA of typical imprints (*Snrpn*, *Igf2r*, *Dlk1/Gtl2* and *Mest*) of the tail tissue from the offspring derived from ESC- and pESC-PGCLC derived mice. PCR products were either digested (D) or undigested (U) with the respective enzyme. The digested and undigested fragments are indicated by black and white triangles, respectively (n = 8). (**E**) Methylation level analysis of COBRA by Image J (n = 8).

Table S1. DNA methylation level of known imprints (100% as 1.000)

Gene Name	Type	ESC-1	ESC-2	pESC-1	pESC-2	E9.5 PGC-1	E9.5 PGC-2	E12.5 PGC-1	E12.5 PGC-2	ESC-PGCLC-1	ESC-PGCLC-2	pESC-PGCLC-1	pESC-PGCLC-2
Peg3	Maternal	0.7473	0.5978	0.7450	0.6879	0.8487	0.8378	0.4723	0.4767	0.7024	0.6851	0.6539	0.6583
Ndn	Maternal	0.3672	0.2536	0.3697	0.3165	0.5889	0.6094	0.3906	0.4047	0.6348	0.7073	0.4330	0.4044
Magel2	Maternal	0.3613	0.3016	0.3335	0.3436	0.5668	0.5536	0.3477	0.3703	0.4598	0.4337	0.4014	0.4000
Snrpn	Maternal	0.3447	0.4481	0.8284	0.8318	0.5921	0.6646	0.4172	0.4813	0.4848	0.5671	0.6616	0.6999
Inpp5f	Maternal	0.3055	0.2814	0.4872	0.5097	0.6097	0.5670	0.3361	0.4186	0.4716	0.4407	0.4728	0.4830
Impact	Maternal	0.2688	0.3609	0.5308	0.5770	0.6194	0.6391	0.4442	0.4489	0.5091	0.5064	0.5269	0.5190
H13	Maternal	0.2174	0.1948	0.1479	0.2684	0.6301	0.6049	0.4081	0.4047	0.4672	0.5238	0.3780	0.3879
Blcap	Maternal	0.1350	0.1160	0.1817	0.2473	0.4447	0.5053	0.2759	0.2617	0.3212	0.3450	0.3071	0.2971
Grb10	Maternal	0.1147	0.0761	0.1472	0.1369	0.2997	0.3173	0.1856	0.1965	0.2139	0.1878	0.1998	0.2004
Slc38a4	Maternal	0.1080	0.0909	0.0447	0.1045	0.4154	0.3861	0.2615	0.1978	0.2980	0.2292	0.2185	0.2508
Mest	Maternal	0.0673	0.0676	0.4763	0.4475	0.4856	0.4899	0.3722	0.2878	0.3447	0.3672	0.4515	0.3872
Mkrn3	Maternal	0.0640	0.0383	0.0516	0.0545	0.5049	0.5201	0.3659	0.3041	0.6595	0.3607	0.3327	0.3027
Igf2	Maternal	0.0578	0.0436	0.0438	0.0488	0.0239	0.0229	0.0215	0.0252	0.0285	0.0273	0.0330	0.0292
Herc3	Maternal	0.0575	0.0629	0.1216	0.1600	0.1629	0.1967	0.0805	0.1155	0.1256	0.1252	0.1206	0.1257
Gnas	Maternal	0.0550	0.0390	0.0820	0.0963	0.4875	0.4753	0.2608	0.3406	0.3395	0.2938	0.2960	0.3173

Sgce	Maternal	0.0325	0.0336	0.3930	0.3543	0.5283	0.5596	0.2856	0.3244	0.3665	0.3359	0.4271	0.4079
Peg10	Maternal	0.0325	0.0352	0.3892	0.3465	0.5092	0.5483	0.2773	0.3147	0.3573	0.3292	0.4175	0.4047
Jade1	Maternal	0.0245	0.0340	0.0356	0.0344	0.2926	0.3213	0.1958	0.2292	0.2178	0.1885	0.1927	0.1795
Mcts2	Maternal	0.0166	0.0376	0.0613	0.0376	0.5885	0.6150	0.3718	0.3949	0.4394	0.4331	0.3572	0.3810
Commd1	Maternal	0.0159	0.0176	0.3143	0.2829	0.3812	0.3848	0.2149	0.2149	0.2393	0.2414	0.2762	0.2664
Peg12	Maternal	0.0137	0.0157	0.0107	0.0200	0.1808	0.1866	0.1097	0.1378	0.1342	0.1201	0.1082	0.1145
Plagl1	Maternal	0.0061	0.0149	0.2605	0.2128	0.5348	0.5965	0.2850	0.3771	0.3235	0.3243	0.3787	0.3770
Nlrp2	Paternal	0.3456	0.2041	0.2766	0.2654	0.4714	0.5343	0.3313	0.3691	0.4120	0.3843	0.3317	0.3614
Slc22a18	Paternal	0.1652	0.1613	0.0871	0.1687	0.6660	0.6612	0.3920	0.4075	0.5505	0.5252	0.4363	0.4083
Igf2r	Paternal	0.1320	0.0950	0.1013	0.1501	0.2538	0.1971	0.1790	0.1318	0.1338	0.1580	0.1141	0.1198
Cdkn1c	Paternal	0.0362	0.0343	0.0295	0.0518	0.2756	0.2811	0.1657	0.1826	0.2041	0.1969	0.1850	0.1633
Kcnq1	Paternal	0.0344	0.0325	0.0231	0.0250	0.0643	0.0584	0.0327	0.0326	0.0406	0.0482	0.0388	0.0474
Rasgrf1	Paternal	0.0152	0.0201	0.0131	0.0215	0.0277	0.0105	0.0183	0.0120	0.0152	0.0147	0.0237	0.0229
Zdbf2	Paternal	0.0151	0.0177	0.0138	0.0136	0.0183	0.0136	0.0150	0.0202	0.0161	0.0160	0.0153	0.0193
Ube3a	Paternal	0.0141	0.0143	0.0113	0.0124	0.0124	0.0138	0.0147	0.0129	0.0118	0.0098	0.0122	0.0124
Dlk1	Paternal	0.0124	0.0172	0.0146	0.0160	0.0183	0.0163	0.0153	0.0168	0.0216	0.0221	0.0181	0.0223

Table S2. Summary of IVM and IVF efficiency

Cell type	No. GV oocytes	No. 2-cell (%)	No. live pups (%)
pESC	181	64 (35.4)	3 (4.7)
ESC	147	52 (35.4)	2 (3.8)
E12.5 PGC[*]	67	22 (32.8)	2 (9.1)
E12.5 PGC[#]	69	49 (71.0)	4 (8.2)

* Collected from OG2 mice (Oct4-GFP, B6CBAF1 background) served as control for *in vivo* PGCs; # Data from Sheng, et al. Protein Cell, 11(12):928-930 (2019). The E12.5 PGCs served as *in vivo* control had the genetic background the same as pESC and ESC (Actin-GFP, B6).

Table S3. Steps of oocyte amplification through parthenogenesis (inbred C57BL/6)

Step 1: MII oocytes to pESC lines			
Group	MII oocytes	pESC lines (%)	pESC lines with GT (%)
Total	32	6 (18.8)	2 (33.3)
**Step 2: pESC proliferation with passages**			
Group	Cell number at P1	Ratio of passage	Cell number at P10
Mean	1.0 x 10^4	1 : 5	9.8 x 10^{10}
**Step 3: PGCLCs derivation**			
Group	No. pESCs	No. PGCLCs	Efficiency (%)
1	1 x 10^5	6.2 x 10^4	62.0
2	1 x 10^5	6.7 x 10^4	67.0
3	1 x 10^5	6.4 x 10^4	64.0
4	1 x 10^5	5.8 x 10^4	58.0
5	1 x 10^5	6.6 x 10^4	66.0
Mean	1 x 10^5	6.3 x 10^4	63.0
**Step 4: rOvary formation (2x10^4 PGCLCs and 10x10^4 somatic cell per Aggregates)**			
Group	Aggregates	rOvaries	Efficiency (%)
Total	30	19	63.3
**Step 5: GV oocytes per rOvary**			
Group	GV oocytes	No. rOvaries	Efficiency
1	44	3	14.7
2	27	3	9.0
3	37	3	12.3
4	37	3	12.3
5	36	3	12.0
Total	181	15	12.1

GT, germline competence; GV oocytes, germinal vesicle oocytes; rOvaries, reconstituted ovaries.

Table S4. Primers for microsatellite genotyping

Gene	Forward	Reverse
D12Mit136	TTTAATTTTGAGTGGGTTTGGC	TTGCTACATGTACACTGATCTCCA
D8Mit94	GTTGGGGCTCTGCTCTCTC	CACATATGCATACATATACATACACGT

Table S5. Primers for Real-Time qPCR

Gene	Forward	Reverse
H19	CTCAGACGGAGATGGACGA	CGATTGCACTGGTTTGGA
Igf2	GGGCGGCTATTGTTGTTGTTCTCA	GGATCCCAGAACCCGAGAAGA
Igf2r	GGGGGCTCCTGGGTAAATGTCAT	AGGCCGGTCGGGATGGAGAG
Snrpn	GCGGGTACTGGGTTGGGGCTC	AGGCCCATCCCAGCAGGTCAT
Impact	GCGCCACCCACAACATCTATGC	AGCGGGACACCACCACCATGAC

Table S6. Primers for COBRA

Gene	Forward	Reverse
H19/Igf2-Outside	GGTTTTTTGGTTATTGAAT TTTAAAAATTAG	AAAAACCATTCCCTAAAATAT CACAAATACC
H19/Igf2-Inside	TTAGTGTGGTTTATTATAG GAAGGTATAGAAGT	TAAACCTAAAATACTCAAAC TTTATCACAAC
Snrpn	AATTTGTGTGATGTTTGTA ATTATTTGG	ATAAAATACACTTTCACTACT AAAATCC
Igf2r-Outside	CACTTTTAAACTTACCTCT CTTAC	TAGAGGATTTTAGTATAATTT TAA
Igf2r-Inside	GAGGTTAAGGGTGAAAAG TTGTAT	CACTTTTAAACTTACCTCTCT TAC
Mest	TTTTAGATTTTGAGGGTTT TAGGTTG	AATCCCTTAAAAATCATCTTT CACAC
Dlk1/Gtl2-Outside	TTAAGGTATTTTTTATTGAT AAAATAATGTAGTTT	CCTACTCTATAATACCCTATAT AATTATACCATAA
Dlk1/Gtl2-inside	TTAGGAGTTAAGGAAAAG AAAGAAATAGTATAGT	TATACACAAAAATATATCTAT ATAACACCATACAA
